# Day Temperature Has a Stronger Effect Than Night Temperature on Anthocyanin and Flavonol Accumulation in ‘Merlot’ (*Vitis vinifera* L.) Grapes During Ripening

**DOI:** 10.3389/fpls.2020.01095

**Published:** 2020-07-24

**Authors:** Yifan Yan, Changzheng Song, Luigi Falginella, Simone D. Castellarin

**Affiliations:** ^1^ Wine Research Centre, The University of British Columbia, Vancouver, BC, Canada; ^2^ Research Center, Vivai Cooperativi Rauscedo, Rauscedo, Italy

**Keywords:** abiotic stress, anthocyanins, flavonols, flavonoids, viticulture

## Abstract

Flavonoids impart color and mouthfeel to grapes and wine and are very sensitive to environmental conditions. Growth chamber experiments were performed to investigate the effect of temperature regimes and the differences between day/night temperatures on anthocyanins and flavonols in Merlot grapes. Among the regimes tested, the ones with diurnal 20°C determined the highest levels of anthocyanins and flavonols. Higher diurnal temperatures decreased those levels but increased the proportion of methoxylated and acylated species. When regimes with the same day temperature but different night temperatures were compared, differences between day/night temperatures did not affect anthocyanins, unless a difference of 25°C between day and night temperatures was imposed. When regimes with the same night temperature but different day temperatures were compared, the regime with higher day temperature had a lower anthocyanin level. No relationships were observed between the effects of temperature regimes on anthocyanin level and the expression of key anthocyanin genes. However, the effects on anthocyanin acylation level were consistent with the effects on the acyltransferase expression, and the effects on flavonol level were consistent with the effects on the expression of key flavonol genes. This study indicates that, in Merlot grapes, anthocyanins and flavonols are mostly sensitive to day temperatures.

## Introduction

Flavonoids constitute the major portion of phenolic compounds in grapes and include anthocyanins and flavonols, as well as flavan-3-ols and proanthocyanidins (also known as tannins) (Teixeira et al., 2013). In red grape varieties, anthocyanins are produced in the berry skin during ripening, act as attractants for seed dispersal, and determine the color of the wines (Shirley, 1996). Flavonols are largely accumulated during blooming and ripening, and function as UV protectors (Carbonell-Bejerano et al., 2014). In red wines, they can co-pigment with anthocyanins to affect color stability, while in white wines, they directly affect the yellowish color (Castillo-Muñoz et al., 2007). Moreover, anthocyanins and flavonols act as antioxidants and have health benefits when consumed, such as cardiovascular disease prevention, obesity control, and diabetes alleviation (He and Giusti, 2010).

Anthocyanins are classified into different subgroups based on their chemical structures: as 3′4′- and 3′4′5′-substituted, according to the number of hydroxyl groups on the B-ring, and as methoxylated or non-methoxylated, according to the presence or absence of methoxyl groups. Likewise, flavonols are classified into 3′-substituted, 3′4′-substituted, and 3′4′5′-substituted, or methoxylated and non-methoxylated according to the same criteria as described above. In grapes, anthocyanins and flavonols are normally accumulated as stable glycosylated forms (Castillo-Muñoz et al., 2007). Furthermore, the 6′-hydrogen on the sugar moiety can be substituted by aliphatic or aromatic acids to generate acylated anthocyanins and flavonols (Mazza and Miniati, 1993). An increased number of hydroxyl groups on the B-ring shifts the anthocyanin color from red to blue, while methoxylation and acylation shift the color to red and blue, respectively (Lachman and Hamouz, 2005; Liu et al., 2018). Generally, glycosylation, methoxylation, and acylation increase the thermal stability of flavonoids (Jackman and Smith, 1996).

The accumulation of anthocyanins and flavonols during berry development is greatly affected by environmental factors (Mori et al., 2007; Matus et al., 2009; Azuma et al., 2012), which suggests that their levels and profiles change among seasons and wine regions, and may also be affected by the predicted climatic changes (Jones et al., 2012).

Among the environmental factors, temperature has arguably the most profound effect on anthocyanin accumulation in grapes (Jones et al., 2012). Early studies showed that high day and night temperature regime (37/32°C) completely inhibited coloration in Emperor grapes (Kliewer, 1977) and that high day temperature (35°C) strongly reduces anthocyanin concentration, while the effect of night temperature changes in relation to the day temperature considered (Kliewer and Torres, 1972). Temperature effects on anthocyanin accumulation also depend on the developmental stages when the berries experience a particular temperature. Yamane et al. (2006) suggested that the most sensitive stage for grape berry to temperature treatment was 1 to 3 weeks after the onset of ripening, defined by viticulturists as veraison (shift of berry color from green to yellow/red). A recent study by Gaiotti et al. (2018) indicated that grapes exposed to cool night temperatures (10–11°C, compared with 15–20°C in control) from 12 days before veraison to the end of veraison enhanced anthocyanin accumulation but only in one out of the two seasons. Anthocyanin composition was also altered by temperature regimes, with the methoxylated and acylated proportion being increased by high temperature treatments (Mori et al., 2005; Tarara et al., 2008; De Rosas et al., 2017).

In contrast to anthocyanins, flavonol accumulation was originally reported to be unaffected by temperatures (Spayd et al., 2002; Azuma et al., 2012). However, recent studies showed that high temperatures (30–40°C) during berry ripening exerted negative effects on flavonol accumulation (Degu et al., 2016; Pastore et al., 2017b). Exposure to high temperatures affected the various flavonols differently: for instance, an increase of 6 or 9°C from ambient temperature caused the complete absence of kaempferol 3-O-glucoside, an 85% reduction of quercetin 3-O-glucoside, and a 65% reduction of myricetin 3-O-glucoside (Pillet, 2011).

It remains unclear whether temperature affects the synthesis of flavonoids directly or whether high temperatures promote degradative events (Mori et al., 2007). Though several studies reported that the expression of phenylpropanoid and flavonoid genes [including *phenylalanine ammonia lyase* (*VviPAL*), *chalcone synthase* (*VviCHS*), *chalcone isomerase* (*VviCHI*), *UDP-glucose: flavonoid 3-O-glucosyltransferase* (*VviUFGT*), and the transcription factors (TFs) *VviMybA1* and *VviMybA2*], were down-regulated by high temperatures (30–35°C, compared with 15–20°C) (Yamane et al., 2006; Azuma et al., 2012; Movahed et al., 2016), other studies reported that the expression of flavonoid genes (*VviUFGT* and *VviMybA*) was not affected (Mori et al., 2007) or up-regulated by high temperatures (Rienth et al., 2014a), yet decreased levels of anthocyanins were observed. Mori et al. (2007) reported a reduced anthocyanin concentration in berries exposed to a high temperature condition (35°C, compared with 25°C) despite similar *VviUFGT* expression and higher UFGT enzyme activity. This indicates that the lower anthocyanin level under high temperature conditions might result from a higher degradation rate rather than a lower biosynthesis rate.

Cohen et al. (2008) and Gaiotti et al. (2018), as well as anecdotal knowledge from the industry, suggest that the difference between day/night temperatures affects the flavonoid concentration in grapes, with greater differences promoting flavonoid concentration. However, those experiments did not provide definitive evidence, and a large knowledge gap remains on the role of night temperature and the difference between day/night temperatures in anthocyanin and flavonol accumulation. This topic is even more important nowadays since the increase of night temperatures due to climate change in the past half century was faster than that of day temperatures, which suggests a further decrease in the difference between day/night temperatures in future years (Stocker et al., 2014).

In this study, growth chamber experiments that compared a wide range of temperature regimes were performed to assess the effect of temperatures on anthocyanins and flavonols in Merlot grapes, with a focus on the impact of the difference between day/night temperatures. Five temperature regimes imposed from veraison to harvest, which ranged from 20 to 35°C during the day, from 5 to 30°C at night, and from 5 to 25°C as a day/night difference, were compared in each experiment. The goal of our research was to identify the optimum temperature for anthocyanin and flavonol accumulation in Merlot grapes, as well as to determine if the temperature difference between day/night affected this accumulation. We hypothesized that a larger difference between day/night temperatures and a lower night temperature might be beneficial for anthocyanin and flavonol accumulation in grapes. Gene expression analysis was also carried out in order to assess the extent of temperature effects on transcriptional regulation of anthocyanin and flavonol synthesis during day and night.

## Materials and Methods

### Experimental Design and Plant Material

Three experiments were conducted in three consecutive years from 2017 to 2019. All three experiments considered own-rooted grapevines of the same variety and clone (*Vitis vinifera* L. cv. Merlot, cl. 347), and utilized five growth chambers.

#### Experiment 1

In 2017, Merlot fruiting cuttings were obtained according to Mullins and Rajasekaran (1981) with some modifications. Three-node mature canes were collected during the dormant season from a commercial vineyard located in the Okanagan Valley, British Columbia, Canada (49°25′ N, 119°56′ W), in February 2017. Cuttings were placed within a heat-controlled container (25°C at the base), at 4°C in darkness; at the bottom of the container, rooting was induced using the ROOTS^®^ Liquid Stimulator (Wilson, Canada) with Greenhouse Potting Mix (Grower’s Nursery Supply Inc, Salem, USA), perlite, and sand (v/v, 3:1:0.5). Afterwards, pre-rooted cuttings were transferred into 7.5 L pots containing Greenhouse Potting Mix and moved to the greenhouse. Vines were grown at 22.5 ± 0.9/19.8 ± 0.5 (day/night temperature, °C) and 54.5 ± 1.6% relative humidity (RH). Environmental solar radiation was supplemented with Greenpower LED (Phillips, Canada) top lighting in order to guarantee 200 µmol m^−2^ s^−1^ of Photosynthetically Active Radiation (PAR) at the table surface for 16 h per day. Plants were irrigated on alternate days and supplemented with a nutrition solution (Relab Den Haan, Hoorn, Netherlands) twice per week in order to avoid any water deficit or nutrient shortage. One shoot per plant was retained and topped after inflorescence development; one cluster per plant was selected at fruit set, while the other clusters were removed. One lateral shoot was left to support the cluster (Mullins and Rajasekaran, 1981).

At 60 days after anthesis (DAA), vines with approximately 50% of the berries showing partial or full red pigmentation (veraison) were selected. Shoot tips were topped to 20 primary leaves and grapevines were transferred into Conviron BDR 16 (Conviron, Winnipeg, Canada) growth chambers. Each chamber contained 12 plants that were divided into four biological replicates. The temperature regimes applied were: i) 20/10 (day/night temperature, °C); ii) 20/15; iii) 25/15; iv) 35/25; v) 35/30. For the sake of simplicity, they were categorized as low (20/10 and 20/15), intermediate (25/15), and high (35/25 and 35/30) temperature regimes (**Table S1**), according to the diurnal temperature. These regimes were chosen to generate two day/night temperature differences: ΔT = 5°C (20/15 and 35/30) and ΔT = 10°C (20/10, 25/15, and 35/25). Two intermediate 1-h stages were set during the day/night and the night/day transitions in order to mimic gradual temperature decrease/increase. The day/night cycle was 14 h/8 h (plus the two 1-h transition stages). Mean PAR at the pot level was set at 400, 100, and 0 µmol m^−2^ s^−1^ in all chambers during the day, transition, and night stages, respectively. Plants were irrigated the same way as in greenhouse and new secondary shoots were removed through the duration of the experiment. Light intensity around the clusters was measured with a PAR sensor (Kipp and Zonen, Delft, Holland); RH was measured with a humidity probe (Campbell Scientific, Edmonton, Canada); berry surface temperature was measured with an infrared thermal gun (NAPA, Canada).

Berry sampling was carried out at five time-points throughout ripening. Eight berries per biological replicate were randomly collected at 62, 76, 89, 104, and 118 DAA (harvest date) at 4 PM for metabolites and gene expression analyses. Additionally, at 76 and 89 DAA (mid-ripening stages), berry samples were also collected at 4 AM, in order to investigate whether diurnal and nocturnal conditions affected flavonoid gene expression. Berries were removed from clusters by carefully cutting the pedicel, immediately flash-frozen in liquid nitrogen, and stored at −80°C until analysis. By the end of the experiment < 18% of the berries had been removed from the clusters.

#### Experiment 2

In 2018, one-year Merlot rooted cuttings obtained from the previous season were pruned to five to six dormant buds on a single cane and grown in 11.25 L pots filled with Greenhouse Potting Mix. Two shoots with two to three clusters each were maintained per vine after fruit-set; the other shoots were removed. The vines were grown in the greenhouse under the same conditions as in Experiment 1 until veraison (69 DAA), when the primary shoots were topped to 20 leaves, and 20 vines at a similar developmental stage were moved into growth chambers (four biological replicates per chamber, one plant per replicate). The five treatments considered were: i) 20/10 (day/night temperature, °C); ii) 20/15; iii) 25/15; iv) 25/20; v) 30/20. They were categorized according to the diurnal temperature as low (20/10 and 20/15), intermediate (25/15 and 25/20), and high (30/20) temperature regimes (**Table S1**). These regimes were chosen to generate two day/night temperature differences: ΔT = 5°C (20/15 and 25/20) and ΔT = 10°C (20/10, 25/15, and 30/20). Day/night cycles, light intensities, and irrigation strategies were the same as described for Experiment 1. Secondary shoots were kept during the experiment. Light, temperature, and RH were measured as described in Experiment 1; berry surface temperature was not recorded in Experiment 2.

Samples of 20 berries were randomly collected from each biological replicate at 79, 93, 113, and 135 DAA at 4 PM, and at 93 and 113 DAA at 4 AM. Berry samples were collected and stored as described in Experiment 1. An additional sample was collected from four randomly selected grapevines out of the 20 used for the experiment at 69 DAA, before the grapevines were moved into growth chambers. By the end of the experiment <20% of the berries had been removed from the clusters.

#### Experiment 3

In 2019, one-year Merlot rooted cuttings obtained from the previous season (2018) were grown in the greenhouse as described in Experiment 2 and 20 vines at a similar developmental stage were moved into growth chambers (four biological replicates per chamber, one plant per replicate) at veraison (67 DAA). The five temperature regimes tested were: i) 20/5 (day/night temperature, °C); ii) 20/15; iii) 30/5; iv) 30/15; v) 30/25. They were categorized according to the diurnal temperature as low (20/5 and 20/15) and high (30/5, 30/15, and 35/25) temperature regimes (**Table S1**). These regimes were chosen to generate three day/night temperature differences: ΔT = 5°C (20/15 and 30/25), ΔT = 15°C (20/5 and 30/15), and ΔT = 25°C (30/5). Growth chamber conditions and irrigation strategies were the same as in Experiment 1 and 2. Secondary shoots were kept during the experiment. Light, temperature, and RH were measured as described in Experiment 1. Samples of 20 berries were collected—as described in Experiment 2—at harvest (113 DAA at 4 PM).

### Berry Sample Preparation

The frozen berry samples were weighed, peeled, and deseeded using a scalpel and tweezers. Berry tissues were kept frozen using liquid nitrogen. Skin, flesh, and seed weights were recorded. The skin and the flesh were ground into fine powder under liquid nitrogen using a mortar and pestle and an electronic mill (A11 S001, IKA Inc., Wilmington, USA), respectively. The skin powder was used for anthocyanin and flavonol quantification (in Experiments 1, 2, and 3) and gene expression analysis (in Experiment 1). The flesh powder was used for the determination of total soluble solids (TSS) and titratable acidity (TA).

### Berry Total Soluble Solids and Titratable Acidity

An aliquot of 2 g flesh powder was thawed at room temperature for 20 min, and the juice was collected by centrifugation at 10,000*g* for 10 min. Berry TSS were measured with a digital refractometer (Reichert A2R200, Reichert GmbH, Seefeld, Germany) and reported as °Brix; TA was measured by titration with 0.1 N NaOH using a 50-ml alkali burette until pH 8.2 and expressed as tartaric acid equivalents (g/L).

### Anthocyanin and Flavonol Analysis

Anthocyanins and flavonols were extracted with aqueous acidified methanol (v/v, methanol:water:formic acid, 49.5:49.5:1) and analyzed by high-performance liquid chromatography ad mass spectrometry (HPLC-MS), according to Downey and Rochfort (2008) with slight modifications. Approximately 0.15 g of skin powder was extracted in 1.5 ml of the extraction solution for 20 min using a sonicator (FS20H, Fisher Scientific, Ottawa, Canada) followed by 10-min centrifugation at 13,000 g at room temperature. The supernatant was filtered (0.22 µm × 13 mm, PVDF Millex Filter, Sigma-Aldrich, Oakville, Canada) and transferred into an amber vial (Agilent Technologies, Mississauga, Canada) using a syringe (Luer-Lok Tip Syringe, Sigma-Aldrich, Oakville, Canada). The pellet was then extracted for a second time with the same procedures described above. The two fractions of the supernatant were combined. Five µL of the extract was injected into an Agilent 1100 Series LC coupled to an MSD Trap XCT Plus System (Agilent Technologies, Mississauga, Canada) and a Diode Array Detector (DAD, Agilent Technologies, Mississauga, Canada). Chromatographic separation was carried out by an Agilent ZORBAX SB-C18 Column (1.8 µm, 4.6 × 50 mm) (Agilent Technologies, Mississauga, Canada) with the temperature maintained at 67.0°C. The mobile phases were aqueous formic acid (v/v, 98:2; Solvent A) and acetonitrile/formic acid (v/v, 98:2; Solvent B). The LC separation used a binary solvent gradient, with a flow rate of 1.20 ml/min. The gradient conditions were 0.20 min, 5.0% solvent B; 6.00 min, 20.0% solvent B; 9.00 min, 80.0% solvent B; 10.00 min; 90.0% solvent B; 10.10 min, 90.0% solvent B; 11.00 min, 5.0% solvent B; and stopped at 11.50 min. Mass spectra were generated *via* electrospray ionization (ESI) in both positive and negative modes. Compound identification was conducted by i) comparing their retention times with those of standards (3-O-glucosides of cyanidin, peonidin, delphinidin, petunidin, and malvidin, 3-O-glucosides of kaempferol, quercetin, myricetin, isorhamnetin, and syringetin, all from Extrasynthese, Genay, France), ii) matching the mass spectra of identified peaks with anthocyanin and flavonol compounds retrieved from published papers, and iii) comparing their elution order (Mazza et al., 1999; Castillo-Muñoz et al., 2007; Downey and Rochfort, 2008). Uncertain peaks were then verified by MS/MS analysis. Anthocyanin and flavonol quantifications were based on UV-vis spectra at wavelengths of 520 and 353 nm and expressed as malvidin 3-O-glucoside and quercetin 3-O-glucoside equivalents, respectively, as commonly reported (among others, Castillo-Muñoz et al., 2007; Downey and Rochfort, 2008). Calibration curves were constructed using five gradient concentrations of malvidin 3-O-glucoside (1–250 µg/ml) (Extrasynthese, Genay, France) and quercetin 3-O-glucoside (0.5–10 µg/ml) (Extrasynthese, Genay, France) solutions.

### RNA Extraction and Gene Expression Analysis

Samples collected in Experiment 1 at 76 and 89 DAA during the day and at night were used for transcript analysis. Total RNA was extracted from 0.2 g of skin powder using Spectrum™ Plant Total RNA Kit (Sigma-Aldrich, Oakville, Canada) according to the manufacturers’ instructions. The quality and integrity of the extracted RNA was assessed by gel electrophoresis, and the amount was assessed by a spectrophotometer (NanoDrop-1000, Thermo Fisher Scientific, Waltham, USA). One µg of extracted RNA was retrotranscribed using the Invitrogen™ SuperScript™ IV Vilo™ Master Mix (Thermo Fisher Scientific, Waltham, USA) following manufacturers’ instructions.

Transcript abundance was assessed by qRT-PCR on a 7500 Real-Time PCR Systems (Applied Biosystems™, ThermoFisher Scientific, Waltham, USA) according to Wang et al. (2019). Specific primers for the tested genes were reported in **Table S2**. *VviUbiquitin* was chosen as the reference gene as reported in Bogs et al. (2006).

### Statistical Analysis

Statistical analyses were performed using SPSS v23.0 (IBM, New York, USA). A one-way ANOVA test was used to evaluate the effect of temperature regimes on all the parameters assessed within each specific sampling point. Separation of means was performed using an LSD test. A two-way ANOVA test was used to evaluate the effect of the temperature regimes and the sampling time, as well as their interactions on the gene expression level. Linear regression analysis was conducted between TSS and total anthocyanin concentration (expressed as µg/g skin FW) to test the relationship between sugar and anthocyanin accumulation during berry ripening, and an ANCOVA test was conducted to assess the significant differences between the coefficients of the linear regression curves from the above test.

## Results

### Temperature Effect on Berry Development, Sugar, and Acid Accumulation

Berry weight at harvest was generally not affected by the temperature regimes, except for the 25/15 regime in Experiment 1, which had a larger berry than the 35/25 and 35/30 regimes (**Table S3**). Skin weight was generally higher in the low than the high temperature regimes. In Experiment 1, it was higher in the 20/10 and 20/15 berries than in berries from any other regimes; in Experiment 2, it was higher in the 20/10 than the 30/20 berries; while in Experiment 3, it was higher in the 20/15 than the 30/5 berries. No difference in the skin-to-berry weight ratio was found in Experiment 1, while a higher ratio was found in the 20/10 and 20/15 berries than in the 25/15, 25/20, and 30/20 berries in Experiment 2, and in the 20/5 and 20/15 berries than in the 30/15 berries in Experiment 3. Seed weight at harvest was not affected by the temperature regimes.

The effect of the difference between day/night temperatures (ΔT = 5 or 10°C in Experiments 1 and 2, ΔT = 5, 15, or 25°C in Experiment 3) was only observed in the skin-to-berry weight ratio in Experiments 2 and 3. Berries exposed to regimes with the same night temperature had a higher skin-to-berry weight ratio when exposed to a smaller difference between day/night temperatures (*e.g.*, 20/15, ΔT = 5°C, *vs.* 25/15, ΔT = 10°C in Experiment 2; 20/15, ΔT = 5°C, *vs.* 30/15, ΔT = 15°C in Experiment 3).

At harvest, berries exposed to the high temperature regimes generally had lower TSS than the ones exposed to the intermediate and low temperature regimes. TSS difference among the temperature regimes were not significant in Experiment 1, while higher TSS were observed in the 20/15 than the 25/20 berries in Experiment 2 and in the 20/5 and 20/15 berries than in the 30/15 berries in Experiment 3 (**Table 1**). Similar results were observed when the evolution of TSS was considered. In Experiment 1, the 20/15 berries had higher TSS than the 35/30 berries at 104 DAA (**Figure S1A**), while in Experiment 2, the 20/10 and 20/15 berries had higher TSS than the 25/20 berries at 113 DAA (**Figure S1B**).

**Table 1:** Temperature effects on berry total soluble solids (TSS) and titratable acidity (TA) at harvest in Experiments 1, 2, and 3.

Experiment	Temperature regimes (day/night temperature, °C)	TSS (°Brix)^‡^	TA (g/L)
Experiment 1 (118 DAA)*	20/10	19.3 ± 0.6	6.34 ± 0.16 a
	20/15	20.6 ± 1.0	6.14 ± 0.15 a
	25/15	19.9 ± 1.2	5.47 ± 0.12 b
	35/25	19.4 ± 0.8	4.80 ± 0.02 c
	35/30	17.3 ± 0.8	4.75 ± 0.27 c
Experiment 2 (135 DAA)	20/10	20.7 ± 0.9 ab	7.02 ± 0.11 a
	20/15	20.8 ± 0.8 a	5.98 ± 0.16 b
	25/15	20.5 ± 0.2 ab	4.70 ± 0.24 c
	25/20	18.2 ± 1.1 b	5.05 ± 0.50 c
	30/20	19.1 ± 0.5 ab	4.63 ± 0.35 c
Experiment 3 (113 DAA)	20/5	21.5 ± 0.8 a	9.14 ± 0.40 a
	20/15	22.4 ± 1.0 a	7.84 ± 0.57 b
	30/5	20.1 ± 0.3 ab	4.97 ± 0.19 c
	30/15	18.9 ± 0.3 b	4.30 ± 0.26 c
	30/25	20.9 ± 0.9 ab	4.24 ± 0.22 c

*DAA refers to days after anthesis; ^‡^Values reported are the mean ± standard error (SE, n = 4). Different letters indicate significantly different means according to an LSD test (p ≤ 0.05).

At harvest, TA was significantly and consistently affected by the temperature regimes in all experiments. TA level progressively decreased with the increase in temperatures (**Table 1**). This was also clear when the evolution of TA was assessed (**Figures S1C, D** **)**. Soon after treatment applications (16 days in Experiment 1, *i.e.*, 76 DAA; 24 days in Experiment 2, *i.e.*, 93 DAA), the low temperature regimes (*i.e.*, 20/10 and 20/15) displayed higher TA levels than other regimes; this higher level in the low temperature regimes was maintained until harvest.

The difference between day/night temperatures had no effect on TSS. When regimes with the same day but different night temperatures were compared, the difference between day/night temperatures affected TA levels only in the low temperature regimes, with a larger difference between day/night temperatures increased the TA level (*e.g.*, 20/10, ΔT = 10°C *vs.* 20/15, ΔT = 5°C in Experiment 2; 20/5, ΔT = 15°C *vs.* 20/15, ΔT = 5°C in Experiment 3) (**Table 1** **;** **Figures S1C, D** **)**. Opposite results were observed when regimes with the same night, but different day temperatures were compared. TA levels were higher in berries exposed to regimes with a smaller difference between day/night temperatures (*e.g.*, 20/15, ΔT = 5°C, *vs.* 25/15, ΔT = 10°C in Experiments 1 and 2; 20/15, ΔT = 5°C, *vs.* 30/15, ΔT = 15°C in Experiment 3). Similar results were observed during berry ripening (**Figures S1C, D** **)**.

### Temperature Effects on Anthocyanin Levels

**Figure 1:**
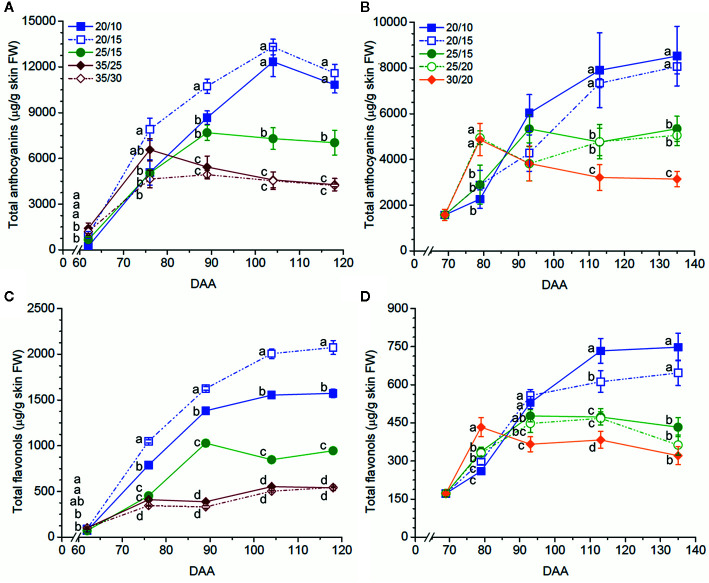
Temperature effects on anthocyanin **(A, B)** and flavonol **(C, D)** concentration (µg/g skin FW) in Merlot grapes in Experiments 1 **(A, C)** and 2 **(B, D)**. Values reported are the mean ± standard error (SE, n = 4). Different letters indicate significant different means according to an LSD test (*p* ≤ 0.05). Legends in **(A)** indicate the temperature regimes in **(A, C)**; legends in **(B)** indicate the temperature regimes in **(B, D)**. DAA refers to days after anthesis.

Anthocyanin levels at harvest were significantly affected by the temperature regimes; the three experiments consistently indicated that the low-temperature regimes and the high-temperature regimes had the highest and the lowest levels, respectively (**Table 2**). Anthocyanins were similarly affected by the temperature regimes during berry ripening, except that the high temperature regimes enhanced anthocyanin levels at early stages of ripening (62 DAA in Experiment 1 and 79 DAA in Experiment 2) (**Figures 1A, B**, **Figures S2A, B**, **Figures S3A, B** **)**.

**Table 2:** Temperature effects on anthocyanin accumulation (concentration, µg/g skin FW) at harvest in Experiments 1, 2, and 3.

Compound*	Anthocyanin concentration (µg/g skin FW)^‡^														
	Experiment 1 (118 DAA)^†^					Experiment 2 (135 DAA)					Experiment 3 (113 DAA)				
	20/10	20/15	25/15	35/25	35/30	20/10	20/15	25/15	25/20	30/20	20/5	20/15	30/5	30/15	30/25
Dp glc	909 ± 56 b	1261 ± 46 a	552 ± 135 c	124 ± 3 d	117 ± 22 d	586 ± 94 a	365 ± 10 b	188 ± 35 bc	271 ± 13 bc	88 ± 8 c	981 ± 150 a	1045 ± 114 a	309 ± 11 b	121 ± 33 b	171 ± 13 b
Cy glc	975 ± 50 a	981 ± 194 a	391 ± 93 b	44 ± 5 c	50 ± 4 c	415 ± 76 a	441 ± 31 a	106 ± 23 b	154 ± 13 b	105 ± 15 b	583 ± 101 a	432 ± 33 a	211 ± 18 b	147 ± 40 b	133 ± 17 b
Pt glc	965 ± 86 a	848 ± 71 a	448 ± 36 b	152 ± 18 c	152 ± 17 c	456 ± 105 a	454 ± 38 a	228 ± 47 b	205 ± 16 b	66 ± 21 c	613 ± 86 b	792 ± 69 a	260 ± 10 c	128 ± 29 c	182 ± 34 c
Pe glc	1945 ± 79 a	2064 ± 107 a	977 ± 47 b	267 ± 25 c	338 ± 40 c	1205 ± 144 a	1223 ± 56 a	628 ± 50 b	560 ± 33 b	208 ± 52 c	1038 ± 145	1251 ± 129	1122 ± 61	1232 ± 160	924 ± 122
Mv glc	2733 ± 80 a	3045 ± 58 a	1728 ± 139 b	995 ± 92 c	1194 ± 17 c	2439 ± 309 a	2482 ± 48 a	1465 ± 171 b	1259 ± 92 b	831 ± 60 c	2558 ± 45 b	3510 ± 209 a	1853 ± 148 c	1304 ± 93 c	1548 ± 186 c
Dp ac-glc	328 ± 45 a	299 ± 14 a	236 ± 76 a	32 ± 2 b	20 ± 2 b	282 ± 87 a	208 ± 9 a	55 ± 14 b	36 ± 9 b	21 ± 6 b	211 ± 24 a	195 ± 39 a	55 ± 7 b	25 ± 7 b	43 ± 10 b
Cy ac-glc	141 ± 9 a	150 ± 30 a	95 ± 32 a	27 ± 3 b	19 ± 1 b	81 ± 25 a	58 ± 7 ab	25 ± 5 b	21 ± 3 b	16 ± 4 b	87 ± 24 a	78 ± 12 a	32 ± 4 b	30 ± 14 b	20 ± 5 b
Pt ac-glc	260 ± 41 a	176 ± 19 b	147 ± 24 b	46 ± 3 c	30 ± 4 c	186 ± 60 a	101 ± 6 ab	57 ± 11 b	37 ± 9 b	23 ± 5 b	165 ± 21 a	176 ± 35 a	67 ± 5 b	36 ± 7 b	53 ± 9 b
Pe ac-glc	304 ± 4 a	333 ± 24 a	245 ± 41 a	71 ± 13 b	66 ± 8 b	389 ± 48 a	324 ± 21 a	180 ± 9 b	126 ± 26 b	21 ± 3 c	207 ± 26 b	250 ± 45 ab	332 ± 26 ab	404 ± 90 a	276 ± 30 ab
Mv ac-glc	1018 ± 112	1044 ± 62	961 ± 69	1205 ± 119	1081 ± 41	1056 ± 129	1004 ± 68	1022 ± 65	965 ± 77	795 ± 31	862 ± 71	1097 ± 113	1129 ± 130	771 ± 50	1032 ± 169
Dp cou-glc	115 ± 22a	104 ± 11 a	87 ± 17 ab	47 ± 10 bc	33 ± 3 c	276 ± 30 ab	367 ± 33 a	249 ± 20 b	181 ± 16 bc	103 ± 21 c	82 ± 8 a	91 ± 17 a	35 ± 4 b	28 ± 5 b	32 ± 5 b
Cy cou-glc	93 ± 8 ab	120 ± 15 a	71 ± 15 b	15 ± 2 c	10 ± 1 c	47 ± 10 ab	50 ± 7 a	31 ± 7 ab	38 ± 9 ab	14 ± 4 b	57 ± 14 a	59 ± 13 a	38 ± 3 b	39 ± 11 b	26 ± 6 b
Pt cou-glc	98 ± 18	88 ± 7	58 ± 6	91 ± 16	60 ± 5	74 ± 13 a	54 ± 2 ab	43 ± 7 ab	35 ± 9 b	29 ± 5 b	77 ± 5 a	86 ± 13 a	61 ± 6 ab	32 ± 3 b	45 ± 5 b
Pe cou-glc	258 ± 7 a	306 ± 25 a	299 ± 36 a	86 ± 13 b	83 ± 6 b	286 ± 72 a	271 ± 13 a	244 ± 37 a	286 ± 63 a	169 ± 18 b	171 ± 18 c	238 ± 40 bc	371 ± 36 ab	437 ± 82 a	306 ± 53 abc
Mv cou-glc	694 ± 102 b	775 ± 76 b	738 ± 47 b	1080 ± 122 a	971 ± 39 a	746 ± 116	661 ± 42	823 ± 52	882 ± 77	652 ± 91	636 ± 77 d	946 ± 30 b	1272 ± 91 a	715 ± 39 cd	858 ± 88 bc
Total	10837 ± 551 a	11594 ± 576 a	7033 ± 798 b	4282 ± 400 c	4223 ± 92 c	8523 ± 1303 a	8061 ± 323 a	5344 ± 547 b	5058 ± 478 b	3143 ± 333 c	8326 ± 442 a	10245 ± 851 a	7147 ± 434 b	5447 ± 572 c	5647 ± 565 c
3′4′-sub	34.5% a	33.9% a	29.4% b	11.9% c	13.3% c	28.4% a	29.3% a	22.7% b	23.3% b	16.7% c	25.4% bc	22.4% c	29.6% b	41.2% a	29.9% b
3′4′5′-sub	65.5% c	66.1% c	70.6% b	88.1% a	86.7% a	71.6% c	70.7% c	77.3% b	76.7% b	83.3% a	74.6% ab	77.6% a	70.4% b	58.8% c	70.1% b
Meth	76.4% b	75.0% b	80.6% b	93.2% a	94.1% a	80.5% c	81.6% c	88.0% a	86.1% b	89.1% a	76.4% b	81.6% b	90.4% a	93.2% a	92.3% a
Non-meth	23.6% a	25.0% a	19.4% a	6.8% b	5.9% b	19.5% a	18.4% a	12.0% c	13.9% b	10.9% c	23.6% a	18.4% a	9.6% b	6.8% b	7.7% b
Non-acy	69.7% a	70.7% a	58.3% b	37.1% d	43.8% c	60.1% a	61.7% a	48.7% b	48.7% b	41.2% c	69.1% a	68.8% a	52.7% b	53.8% b	52.5% b
Ac-glc	18.8% d	17.3% d	23.7% c	32.3% a	28.8% b	23.2% bc	21.0% c	25.2% b	23.4% bc	28.3% a	18.5% b	17.3% b	22.4% a	23.2% a	25.1% a
Cou-glc	11.5% d	12.0% d	18.0% c	30.6% a	27.4% b	16.7% c	17.3% c	26.1% b	27.9% b	30.5% a	12.4% b	13.9% b	24.9% a	23.0% a	22.4% a

* Dp, Cy, Pt, Pe, Mv refer to cyanidin, peonidin, petunidin, peonidin, and malvidin, respectively; glc refers to 3-O-glucoside, ac-glc refers to 3-O-(6′′-acetyl)glucosides, and cou-glc refers to 3-O-(6′′-p-coumaroyl)glucosides; 3′4′-sub, 3′4′5′-sub, Meth, Non-meth, Non-acy, Ac-glc, and Cou-glc refer to the relative concentration of total 3′4′-substituted anthocyanins, 3′4′5′-substituted anthocyanins, methoxylated anthocyanins, non-methoxylated anthocyanins, 3-O-monoglucosides, 3-O-(6′‘-acetyl)glucosides, and 3-O-(6′′-p-coumaroyl)glucosides to total anthocyanins; ^‡^Anthocyanin peaks were integrated by UV-vis spectra at 520 nm; anthocyanin concentration were expressed as µg/g skin FW based on malvidin 3-O-glucoside equivalents. Values reported are the mean ± standard error (SE, n = 4). Different letters indicate significantly different means within each experiment according to an LSD test (p ≤ 0.05); ^†^DAA refers to days after anthesis.

When regimes with the same day but different night temperatures were compared, the difference between day/night temperatures had no effect on the anthocyanin level, except in Experiment 3, where the anthocyanin level was higher in the 30/5 than the 30/15 and 30/25 berries (**Table 2**). When regimes with the same night but different day temperatures were compared, a smaller difference between day/night temperatures (*e.g.*, 20/15, ΔT = 5°C, *vs.* 25/15, ΔT = 10°C, in Experiments 1 and 2; 20/15, ΔT = 5°C, *vs.* 30/15, ΔT = 15°C in Experiment 3) was found to increase the anthocyanin level (**Table 2**). Differences between these regimes were remarkable; for example, in Experiment 1, anthocyanin concentration in the 20/15 berries was 39.3% higher than in the 25/15 berries at harvest. Similar results were observed during ripening in Experiments 1 and 2 (**Figures 1A, B**, **Figures S2A, B**, **Figures S3A, B** **)**, with a few exceptions (76 and 89 DAA in Experiment 1).

### Temperature Effects on Anthocyanin Profiles

Fifteen anthocyanins were profiled in Merlot berry skin, namely glucosides, acetyl glucosides, and *p*-coumaroyl glucosides of cyanidin, peonidin, delphinidin, petunidin, and malvidin (**Table 2** and **S4**).

The temperature regimes greatly affected the relative concentration of various anthocyanin subfamilies in each experiment; at harvest, the proportion of methoxylated (peonidin, petunidin, and malvidin) and acylated (acetyl- and *p*-coumaroyl-) anthocyanins was consistently enhanced by the high temperature regimes, while the proportion of 3′4′5′-substituted (delphinidin, petunidin, and malvidin) anthocyanins increased by the high temperature regimes in Experiments 1 and 2 while not in Experiment 3 (**Table 2**). The relative concentration of acetyl and *p*-coumaroyl glucosides increased progressively with the increase in day temperature (**Table 2**). The only exception was the acetyl fraction in Experiment 2, which did not vary among the intermediate- and low-temperature regimes. The effect of the temperature regimes on the anthocyanin profile during ripening was similar to that reported at harvest in Experiment 1 (**Figures S4** and **S5**), while it varied in Experiment 2 (**Figure S5**).

As was observed for total anthocyanin levels, when day temperature was held constant, the difference between day/night temperatures showed no effect on the proportions of methoxylated, acylated, and 3′4′5′-substituted anthocyanins at harvest (**Table 2**), with three exceptions observed. The proportion of acylated (both acetyl- and *p*-coumaroyl-) anthocyanins was higher in the 35/25 than the 35/30 berries in Experiment 1; the proportion of methoxylated anthocyanins was higher in the 25/15 than in the 25/20 berries in Experiment 2; and the proportion of 3′4′5′-substituted anthocyanins was higher in the 30/5 and 30/25 berries than in the 30/15 berries in Experiment 3. When regimes with the same night but different day temperatures were compared, the relative concentrations of methoxylated, acylated, and 3′4′5′-substituted anthocyanins were higher in temperature regimes with a larger difference between day/night temperatures (*e.g.*, 25/15, ΔT = 10°C, *vs.* 20/15, ΔT = 5°C, in Experiments 1 and 2; 30/15, ΔT = 15°C, *vs.* 20/15, ΔT = 5°C in Experiment 3) with two exceptions. No significant differences were observed in the proportion of methoxylated anthocyanins between the 20/15 and 25/15 berries in Experiment 1; the proportion of 3′4′5′-substituted anthocyanins were higher in the 20/5 and 20/15 berries than in the 30/5 and 30/15 berries, respectively, in Experiment 3.

### Temperature Effects on Flavonol Levels

The low temperature regimes consistently promoted flavonol level at harvest in the three experiments; the intermediate temperature regimes caused an intermediate flavonol level in Experiment 1 but comparable levels with those of the high temperature regime in Experiment 2 (**Table 3**). Similar results were observed during berry ripening in Experiments 1 and 2 (**Figures 1C, D** **;** **Figures S2C, D** **;** **Figures S3C, D** **)**.

**Table 3:** Temperature effects on flavonol accumulation (concentration, µg/g skin FW) at harvest in Experiment 1, 2, and 3.

Compound*	Flavonol concentration (µg/g skin FW)^‡^														
	Experiment 1 (118 DAA) †					Experiment 2 (135 DAA)					Experiment 3 (113 DAA)				
	20/10	20/15	25/15	35/25	35/30	20/10	20/15	25/15	25/20	30/20	20/5	20/15	30/5	30/15	30/25
M gal	35 ± 4 a	38 ± 10 a	19 ± 4 b	13 ± 2 b	14 ± 4 b	23 ± 4 a	17 ± 1 ab	9 ± 2 b	9 ± 1 b	17 ± 1 ab	9 ± 2 ab	6 ± 1 bc	5 ± 1 c	10 ± 1 a	12 ± 1 a
M glc	95 ± 13 b	129 ± 12 a	90 ± 14 b	69 ± 10 c	67± 4 c	20 ± 3 a	16 ± 1 ab	9 ± 1 b	12 ± 2 ab	13 ± 2 ab	21 ± 2 a	21 ± 5 a	26 ± 4 a	17 ± 3 a	16 ± 1 a
M glcU	218 ± 14 b	282 ± 23 a	175 ± 19 c	132 ± 24 d	133 ± 16 d	134 ± 8 a	118 ± 6 ab	111 ± 10 bc	90 ± 4 c	75 ± 5 c	133 ± 11 a	125 ± 3 ab	109 ± 3 b	112 ± 3 b	106 ± 6 b
Q gal	96 ± 12 b	115 ± 10 a	38 ± 5 c	27 ± 4 c	11 ± 3 d	29 ± 3 a	26 ± 3 a	8 ± 1 b	7 ± 2 b	8 ± 1 b	53 ± 4 b	62 ± 2 a	11 ± 1 c	10 ± 2 c	10 ± 1 c
Q glcU	194 ± 7 b	292 ± 63 a	126 ± 6 c	59 ± 5 d	48 ± 8 d	141 ± 8 a	125 ± 7 a	53 ± 11 b	55 ± 5 b	29 ± 2 b	173 ± 11 a	195 ± 15 a	137 ± 12 b	113 ± 5 b	103 ± 10 b
Q glc	685 ± 48 b	879 ± 76 a	334 ± 12 c	139 ± 22 d	165 ± 13 d	216 ± 12 a	205 ± 7 a	125 ± 21 b	95 ± 5 b	97 ± 6 b	223 ± 9 a	248 ± 13 a	127 ± 11 b	113 ± 13 b	112 ± 4 b
K ac-glc	45 ± 4 b	70 ± 2 a	47 ± 5 b	26 ± 1 c	23 ± 2 c	58 ± 3 a	51 ± 4 a	38 ± 5 b	31 ± 3 b	20 ± 3 c	38 ± 3 a	36 ± 3 a	25 ± 3 b	19 ± 2 b	17 ± 3 b
K glc	81 ± 9 b	121 ± 11 a	27 ± 6 c	11 ± 2 d	11 ± 2 d	27 ± 2 a	13 ± 2 b	7 ± 2 c	6 ± 1 c	9 ± 2 bc	79 ± 5 a	79 ± 4 a	47 ± 5 b	27 ± 2 c	34 ± 4 c
Q ram-glc	63 ± 11 a	74 ± 14 a	39 ± 6 b	14 ± 3 c	15 ± 4 c	37 ± 5 a	21 ± 3 b	13 ± 3 bc	11 ± 2 c	11 ± 2 c	19 ± 1 a	19 ± 3 a	12 ± 2 b	8 ± 1 bc	6 ± 1 c
I glc	34 ± 2 b	46 ± 2 a	23 ± 3 c	7 ± 1 e	14 ± 2 d	27 ± 6 a	22 ± 2 a	17 ± 3 ab	10 ± 1 b	11 ± 2 b	47 ± 3 b	56 ± 3 a	32 ± 3 c	29 ± 2 c	25 ± 3 c
S glc	26 ± 3 b	29 ± 4 b	29 ± 3 b	44 ± 3 a	44 ± 7 a	38 ± 5	34 ± 4	44 ± 4	36 ± 2	32 ± 1	38 ± 2	34 ± 1	41 ± 2	38 ± 3	41 ± 3
Total	1573 ± 44 b	2074 ± 77 a	946 ± 25 c	541 ± 30 d	545 ± 6 d	748 ± 54 a	646 ± 20 a	433 ± 58 b	363 ± 10 b	321 ± 15 b	835 ± 34 a	882 ± 31 a	571 ± 39 b	497 ± 22 b	482 ± 19 b
4′-sub	8.0%	9.3%	7.8%	6.9%	6.3%	11.4%	9.9%	10.4%	10.3%	8.7%	14.0% a	13.1% a	12.6% a	9.3% b	10.5% b
3′4′-sub	68.2% a	67.7% a	59.2% b	45.6% c	46.4% c	60.1% a	61.6% a	49.0% b	49.1% b	48.7% b	61.9% a	65.7% a	55.5% b	54.9% b	53.2% b
3′4′5′-sub	23.8% c	23.0% c	33.0% b	47.5% a	47.3% a	28.5% b	28.5% b	40.6% a	40.6% a	42.6% a	24.1% b	21.2% b	31.9% a	35.8% a	36.3% a
Meth	3.8% c	3.7% c	5.4% b	9.4% a	10.6% a	8.5% b	8.7% b	14.1% a	12.8% a	13.4% a	10.2% b	10.3% b	12.8% a	13.4% a	13.7% a
Non-meth	96.2% a	96.3% a	94.6% b	90.6% c	89.4% c	91.5% a	91.3% a	85.9% b	87.2% b	86.6% b	89.8% a	89.7% a	87.2% b	86.6% b	86.3% b

* M, Q, K, I, S refer to myricetin, quercetin, kaempferol, isorhamnetin, and syringetin, respectively; glc refers to 3-O-glucoside, gal refers to 3-O-galactoside; glcU refers to 3-O-glucuronide, ac-glc refers to 3-O-(6′′-acetyl)glucoside, and ram-glc refers to 3-O-(6′′-rhamnosyl)glucoside; 4′-sub, 3′4′-sub, 3′4′5′-sub, Meth, and Non-meth refer to the relative concentration of 4′-substituted flavonols, 3′4′-substituted flavonols, 3′4′5′-substituted flavonols, methoxylated flavonols, and non-methoxylated flavonols to total flavonols; ^‡^Flavonol peaks were identified by UV-vis spectra at 353 nm; flavonol concentrations were expressed as µg/g skin FW based on quercetin 3-O-glucoside equivalents. Values reported are the mean ± standard error (SE, n = 4). Different letters indicate significantly different means within each experiment according to an LSD test (p ≤ 0.05); ^†^DAA refers to days after anthesis.

When regimes with the same day but different night temperatures were compared, a higher flavonol level was observed in the low temperature regime with a smaller difference between day/night temperatures (*i.e.*, 20/15, ΔT = 5°C, *vs.* 20/10, ΔT = 10°C) in Experiment 1 but not in Experiments 2 and 3 (**Table 3**). This was observed throughout the season in Experiment 1 (**Figure 1C**, **Figure S2C**, **Figure S3C**). On the other hand, when regimes with the same night but different day temperatures were compared, a smaller difference between day/night temperatures (*e.g.*, 20/15, ΔT = 5°C, *vs.* 25/15, ΔT = 10°C, in Experiments 1 and 2; 20/15, ΔT = 5°C, *vs.* 30/15, ΔT = 15°C in Experiment 3) was found to increase the flavonol level, except that no differences were found between the 25/20 and 30/20 berries in Experiment 2 in general.

### Temperature Effects on Flavonol Profiles

Eleven flavonols were profiled in Merlot berry skin (**Tables 3** and **S4**) in the three experiments. At harvest, the quercetin was the most abundant flavonol (constituting of 55.29%, 50.31%, 52.53% of total flavonols in Experiments 1, 2, and 3, respectively), and was present as glucoside, glucuronide, galactoside, and (rhamnosyl)glucoside (**Table 3**).

The temperature regimes affected the relative concentration of flavonol subfamilies among experiments, with the proportion of methoxylated (isorhamnetin and syringetin) and 3′4′5′-substituted (myricetin and syringetin) flavonols promoted by the high temperature regimes at harvest (**Table 3**). At the same stage, a lower proportion of 3′4′-substituted flavonols was observed in the high temperature regimes, but no differences were observed for the 4′-substituted flavonol proportion among the temperature regimes, with an exception that a lower proportion was observed in two of the high temperature regimes (*i.e.*, 30/15 and 30/25) in Experiment 3. The effect of the temperature regimes on the flavonol profile during ripening was similar as the one reported at harvest in Experiment 1 (**Figures S6** and **S7**), while it varied in Experiment 2 (**Figure S7**).

The difference between day/night temperatures exerted no effect on the relative abundance of methoxylated and 4′-, 3′4′-, or 3′4′5′-substituted flavonols when regimes with the same day but different night temperatures were compared (**Table 3**). When regimes with the same night but different day temperatures were compared, a larger difference between day/night temperatures (*e.g.*, 25/15, ΔT = 10°C, *vs.* 20/15, ΔT = 5°C, in Experiments 1 and 2; 30/15, ΔT = 15°C, *vs.* 20/15, ΔT = 5°C in Experiment 3) was found to enhance the relative concentration of methoxylated and 3′4′5′-substituted flavonols. However, no differences between the 25/20 and 30/20 berries were observed in Experiment 2.

### Temperature Effects on the Expression of Flavonoid Genes

**Figure 2:**
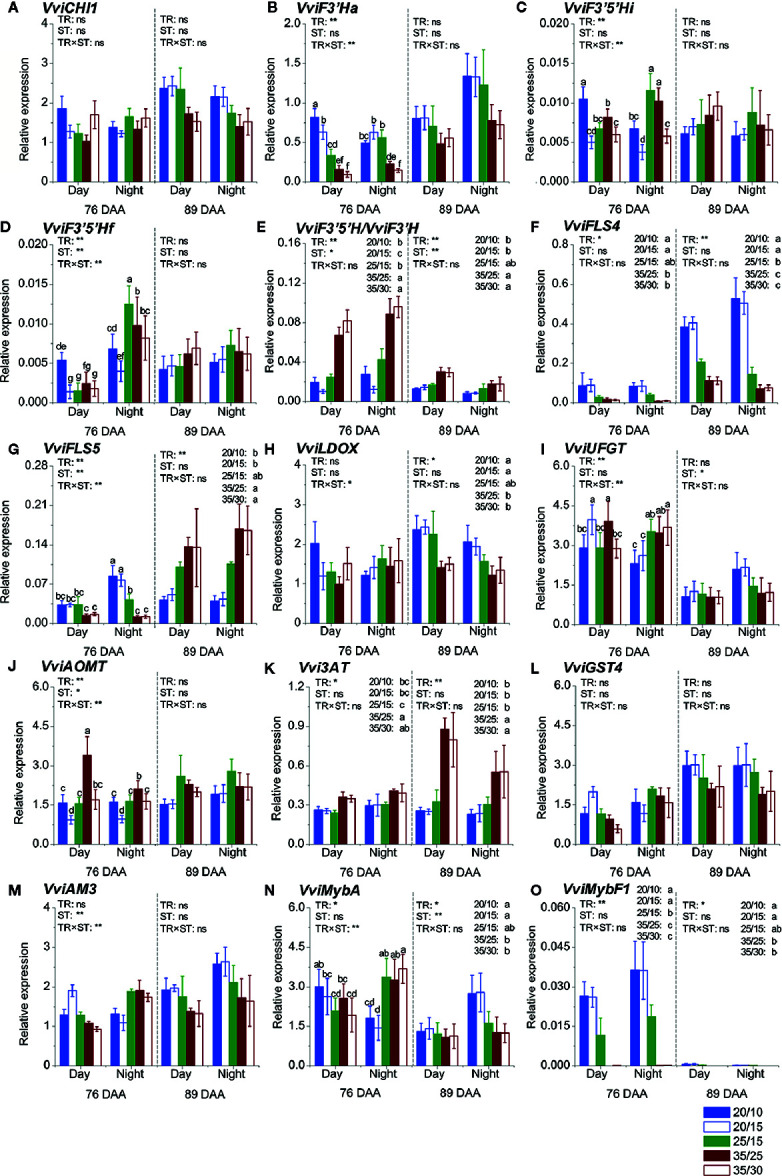
Expression analyses of flavonoid genes in Experiment 1. Expression level of general flavonoid pathway genes **(A–D)**; ratio of *VviF3'5'H to VviF3'H* expression **(E)**; expression level of flavonol biosynthesis genes **(F, G)**; expression level of anthocyanin biosynthesis, modification, and transport genes **(H–M)**; and expression level of flavonoid pathway transcription factors **(N, O)** during the day and night at 76 and 89 DAA. Values were reported as relative expression levels to the expression of the reference gene *VviUbiquitin*. TR and ST indicate temperature regimes and sampling time (day or night), respectively. Values reported are the mean ± standard error (SE, n = 4). Two-way ANOVA was used to assess the effect of temperature regimes, sampling time, and the interaction effect between temperature regimes and sampling time. *, **, and ns stand for *p* ≤ 0.05, *p* ≤ 0.01, and not significant, respectively. Different letters indicate significantly different means at each time point according to an LSD test (*p* ≤ 0.05). DAA refers to days after anthesis.

A set of fourteen genes involved in anthocyanin and flavonol biosynthesis, modification, and transport was selected for assessing the impact of the temperature regimes on the expression of the flavonoid pathway genes during day and night at 76 and 89 DAA in Experiment 1 (**Figure 2**).

Of the fourteen genes, eight did not change their expressions between day/night; on the contrary, *flavonoid 3*′*5*′*-hydroxylase* (*VviF3*′*5*′*Hf*), *flavonol synthase* (*VviFLS5*), *VviUFGT*, *anthoMATE3* (*VviAM3*), and *VviMybA* (**Figures 2D, G, I, M, N** **)** generally showed higher expression at night than during the day, at least at one of the developmental stages tested.

The expression of three genes was not affected by the temperature regimes: *VviCHI1*, *Glutathione S-transferase* (*VviGST4*), and *VviAM3* (**Figures 2A, L, M** **)**. The remaining genes were affected by the temperature regimes, the sampling time (day *vs.* night), and the developmental stage (76 *vs.* 89 DAA) differentially across genes. *Flavonoid 3*′*-hydroxylase* (*VviF3*′*Ha*) (**Figure 2B**) showed a higher expression level in the low temperature regimes, but only at 76 DAA. The impact of the temperature regimes on the expression patterns of the two *VviF3*′*5*′*H* genes tested (*VviF3*′*5*′*Hi* and *VviF3*′*5*′*Hf*) was similar (**Figures 2C, D** **)**. Differences among the temperature regimes were observed at 76 DAA; the highest expression was observed in the 20/10 berries during the day and in the 25/15 and 35/25 berries at night.

The expression of *VviFLS4* was consistently down-regulated by the high temperature regimes during day and night, at both developmental stages (**Figure 2F**), while the expression of the other *VviFLS* tested, *VviFLS5*, was down-regulated by the same regimes at 76 DAA; however, was up-regulated by the high temperature regimes at 89 DAA (**Figure 2G**). The expression of *leucoanthocyanidin dioxygenase* (*VviLDOX*) **(** **Figure 2H**) was not affected by the temperature regimes at 76 DAA but was down-regulated by the high temperature regimes (*i.e.*, 35/25 and 35/30) at 89 DAA.

The expression of *VviUFGT* was affected by the temperature regimes at 76 DAA, with higher levels in the 20/15 and 35/25 berries during the day, and in the 25/15, 35/25, 35/30 berries at night. Despite the trend toward higher expression levels in the 20/15 and 20/10 berries than in the 35/25 and 35/30 berries at 89 DAA at night, the differences were not significant.

The highest and the lowest expression level of *anthocyanin O-methyl transferase* (*VviAOMT*) (**Figure 2J**) was found in the 35/25 and 20/15 berries, respectively, during the day at 76 DAA. Neither the temperature regimes nor the sampling time affected the expression at 89 DAA. The expression of *anthocyanidin 3-O-glucoside 6*′′*-O-acyltransferase* (*Vvi3AT*) (**Figure 2K**) was consistently promoted by the high temperature regimes (*i.e.*, 35/25 and 35/30), particularly at 89 DAA.

Despite an interaction effect between the temperature regimes and the sampling time on the expression level being observed at 76 DAA, the expression of *VviMybA*—a key anthocyanin TF—was generally found to be higher in the low-temperature regimes (*i.e.*, 20/10 and 20/15) during the day, and in the intermediate- and high-temperature regimes (*i.e.*, 25/15, 35/25, and 35/30) at night (**Figure 2N**). At 89 DAA, the expression of *VviMybA* was ~2-fold higher in berries exposed to the low temperature regimes (*i.e.*, 20/10 and 20/15) than in berries exposed to other regimes. The TF that regulates flavonol biosynthesis, *VviMybF1*, was modulated similarly as its target *VviFLS4* (**Figure 2O**).

The difference between day/night temperatures did not affect the expression of most genes when regimes with the same day temperature were compared, except for *VviF3*′*Ha*, *VviF3*′*5*′*Hi*, *VviF3*′*5*′*Hf*, *VviUFGT*, and *VviAOMT*, which were affected at 76 DAA. Generally (except for *VviUFGT*), a larger difference between day/night temperatures (*e.g.*, 20/10, ΔT = 10°C, *vs.* 20/15, ΔT = 5°C) increased the expression level. When regimes with the same night temperature were compared, it was found that a smaller difference between day/night temperatures (*e.g.*, 20/15, ΔT = 5°C, *vs.* 25/15, ΔT = 10°C) increased the expression of *VviFLS4*, *VviLDOX*, and *VviMybF1* at one of the stages at least, while a larger difference between day/night temperatures (*e.g.*, 25/15, ΔT = 10°C, *vs.* 20/15, ΔT = 5°C) increased *Vvi3AT* expression at both stages. The expression of *VviFLS5* was increased in the 20/15 (ΔT = 5°C) berries at 76 DAA, but decreased in the same regime at 89 DAA, compared to the 25/15 berries (ΔT = 10°C).

## Discussion

The negative effect of elevated temperatures on anthocyanin accumulation has been well documented in grapes (Kliewer and Torres, 1972; Mori et al., 2007; Tarara et al., 2008; De Rosas et al., 2017). Our study employs a large range of temperature regimes, and the results indicate that the optimum temperature for anthocyanin accumulation in Merlot berries is around 20°C during the day. Mori et al. (2007) reported that high day temperature (35°C) reduced the total anthocyanin content in Cabernet Sauvignon berries to less than half of that in control (25°C). It is also observed in our study that a 5°C increase in day temperature from 20 to 25°C severely reduces the anthocyanin level (37%, in Experiment 1 and 2). Recently, De Rosas et al. (2017) reported that a small increase in temperature (+2.5–3°C from an average of 24°C in controls) resulted in a ~40% and 28% to 41% loss of anthocyanins in Bonarda and Malbec berries, respectively.

Temperature was often reported to have no effect on flavonol accumulation. However, in our study, it has been observed that flavonol accumulation is strongly inhibited by the high temperature regimes. In addition, there is a reduction in flavonol accumulation in the intermediate temperature regimes as well, compared to the low temperature regimes (*e.g.*, 25/15 in Experiment 1, 25/15 and 25/20 in Experiment 2). Inconsistencies among studies might be attributed to the milder temperature treatments applied (Spayd et al., 2002), different grape varieties studied (Mori et al., 2005), different scales of the study conducted, *e.g.*, field studies (Cohen et al., 2008)—where various environmental factors can interact to temperature—or *in vitro* systems (Azuma et al., 2012)—where cultured tissues might affect cell metabolic responses. In this study, despite the consistent effects of temperature regimes on the total flavonol levels observed across the three experiments, these levels were on average lower in Experiments 2 (−56%) and 3 (−43%) than in Experiment 1. Flavonol biosynthesis in grapes is sensitive to light (Teixeira et al., 2013). Despite light intensity been set at the same level in the growth chambers, incident light measured at the cluster level was lower in Experiments 2 and 3 than in Experiment 1 (**Table S6**), likely determining the lower flavonol levels.

The reduction in berry weight observed with high temperature regimes in Experiment 1 was potentially caused by the higher vapour pressure deficit in the growth chambers with high temperatures (that were also characterized by lower humidity) as in Sweetman et al. (2014). Previous studies showed that higher vapour pressure deficits increase berry transpiration reducing berry water content and size (Zhang and Keller, 2015). In this study, temperature regimes also induced changes in the skin-to-berry weight ratio. Both concentrations and content per berry of anthocyanins and flavonols are similarly affected by the temperature regimes. Thus, differences in berry size and skin-to-berry weight ratio observed among the temperature regimes—which have been reported to affect anthocyanin concentration (Wong et al., 2016)—have a minor role in determining the differences in anthocyanin and flavonol concentration in the current study.

Our study reveals strong effects of high temperatures on anthocyanin and flavonol profiles. Higher relative abundance of methoxylated anthocyanins and flavonols, as well as acylated anthocyanins, are part of the Merlot response to high temperatures. Similarly, relative concentrations of methoxylated and acylated anthocyanins were increased in Malbec and Bonarda grapes grown under high temperatures (2.5–3°C higher than control temperatures) (De Rosas et al., 2017) and in Pinot noir grapes grown under high night temperature (30°C, *vs.* 15°C in control) (Mori et al., 2005).

Methoxylation and acylation have been known to enhance the chemical and thermal stability of anthocyanins (Liu et al., 2018). Therefore, the modification of anthocyanin and flavonol profiles under high temperatures might be promoted by the biosynthesis of more stable forms (*e.g.*, methoxylated anthocyanins and flavonols) with a higher resistance to thermal degradation (Sadilova et al., 2006). However, the altered profiles under the high temperature regimes might also be due to an unpaired degradation that affected more severely the non-methoxylated and non-acylated species.

In this study, caffeoyl glucosides were not detected in Merlot grapes, a consistent finding with several other studies (Tarara et al., 2008; Dimitrovska et al., 2011; Cook et al., 2015; Hilbert et al., 2015; Sivilotti et al., 2016). However, caffeoyl glucosides have been found in Merlot grapes (Mazza et al., 1999; Liang et al., 2014; Shi et al., 2016; Shi et al., 2018). This suggests that growing conditions including environmental factors and viticultural strategies, or the clone chosen may determine the presence and/or the levels of caffeoyl glucosides in Merlot grapes. However, it is worth noting that caffeoyl glucosides are generally a small fraction (0.03–0.77% across 64 red grape varieties) of total anthocyanins (Mattivi et al., 2006), and when present it is unlikely that they would affect the general response of anthocyanins to temperature regimes.

The cultivation systems (*e.g.*, potted vines grown in the growth chamber *vs.* vines grown in open fields) may affect the physiological response of grapes to temperature regimes; therefore, caution is required when interpreting the results of this study. However, in this study, anthocyanin levels and profiles as well as temperature-related changes were similar to the ones reported in open field studies (Tarara et al., 2008).

The expression of *VviUFGT*, which determines anthocyanin accumulation during ripening, is not affected by the temperature regimes in our study. Consistent with this finding, Mori et al. (2007) reported that the transcript abundance of *VviUFGT* was not affected by high temperature (35°C), while the activity of UFGT enzyme was increased, although the accumulation of anthocyanins decreased. These results suggest that the lower anthocyanin level in berries under the high temperature regimes might result from a different mechanism (post-transcriptional regulation or metabolite degradation). Moreover, Lecourieux et al. (2019) revealed that the transcriptome and proteome correlate poorly in heat-stressed berries and highlighted the importance of analysing the proteome for understanding the metabolite responses in heat-stress berries. Unlike with anthocyanins, the down-regulation of key flavonol genes such as *VviFLS4* (also named as *VviFLS1* in Lecourieux et al., 2017 and Sun et al., 2017) and *VviMybF1* in berries under the high temperature regimes matched the decreased level of flavonols, which suggests a transcriptional control of flavonols in response to the temperature regimes. Lecourieux et al. (2017) reported a strong inhibition of *VviFLS4* expression under heat stress during berry ripening, which led to reduced flavonol accumulation. *VviMybF1* is known to be the specific TF of *VviFLS4* (Czemmel et al., 2009; Czemmel et al., 2017), and a synchronous down-regulation of *VviFLS4* and *VviMybF1* has been observed in the high temperature regimes in the present study.

Anthocyanin accumulation in plants depends on the turnover between biosynthesis and degradation (Niu et al., 2017; Liu et al., 2018), and the latter was shown to be enhanced under high temperature conditions (Mori et al., 2007; Movahed et al., 2016; Niu et al., 2017). Mori et al. (2007) reported the degradation of anthocyanins in grape berries incubated at 35°C by isotope tracing. Niu et al. (2017) investigated the biosynthesis and degradation of anthocyanins in plums, showing that even though the activity of the anthocyanin biosynthetic enzymes was increased by high temperature, 79% of anthocyanins were degraded by a class III peroxidase after 9 days of exposure at 35°C. In grapes, a recent study suggested a direct role of peroxidases on anthocyanin catabolism (Movahed et al., 2016). Protocatechuic acid, phloroglucinol acid, and 4-hydroxybenzonic acid have been reported as major degradation products of anthocyanins in black carrot, strawberry, and elderberry extracts subjected to high temperature (Sadilova et al., 2006; Sadilova et al., 2007). In our study, protocatechuic acid and 4-hydroxybenzoic acid, as well as other anthocyanin degradation products such as gallic acid and syringic acid (Yang et al., 2018), were detected in Merlot grapes by LC/MS-MS analysis (**Figure S8**, **Table S5**). However, since the abovementioned phenolic acids are commonly detected in unripe grapes (Eyduran et al., 2015), whether these compounds are accumulated in the berry from anthocyanin catabolism or *via* other pathways requires further investigation.

Despite the weak relationship between the changes induced by the temperature regimes on anthocyanin accumulation and gene expression (data not shown), the up-regulation of *Vvi3AT* matches the increased proportion of acylated anthocyanins observed in berries exposed to the high temperature regimes, suggesting a regulatory mechanism at the transcriptional level. However, the expression pattern of *VviAOMT* does not explain much about the increased methoxylated anthocyanin proportion in the high temperature regimes. This could be explained by a higher stability of the methoxylated forms at high temperatures (Jackman and Smith, 1996; Liu et al., 2018). However, as different methoxylation levels among temperature regimes were established soon after treatment application (**Figure S5C**), the time points chosen for gene expression analysis might have missed the stage when *VviAOMT* was modulated by temperatures, as well as we cannot exclude that other enzymes could have contributed to the final proportion of methoxylated anthocyanins under the different temperature regimes (Fournier-Level et al., 2011; Giordano et al., 2016). The relative concentration of 3′4′5′-substituted anthocyanins and flavonols is higher in the intermediate- and high-temperature regimes with comparison to the low temperature regimes in Experiments 1 and 2, as previously reported (Tarara et al., 2008; Pillet, 2011; Azuma et al., 2012; Pastore et al., 2017a), which matches the increased ratio of the *VviF3*′*5*′*H*s (cumulative expression levels of *VviF3*′*5*′*Hi* and *VviF3*′*5*′*Hf*) to *VviF3*′*Ha*. However, a lower proportion of 3′4′5′-substituted anthocyanins is observed in berries exposed to the high temperature regimes in Experiment 3 at harvest (113 DAA). In previous studies, either an increase or decrease of the 3′4′5′-substituted proportion have been reported in response to high temperatures (Mori et al., 2007; Tarara et al., 2008; Pastore et al., 2017b). These discrepancies may be related to the developmental stages when treatments were applied. When the temperature treatments were applied one week before veraison, high temperature (30°C compared to 20°C) induced a lower proportion of 3′4′5′-substituted anthocyanins in Pinot noir grapes *via* the up-regulation of *VviF3*′*H* expression (Mori et al., 2007). Similar results were reported in Sangiovese grapes exposed to high temperatures from one week before veraison (Pastore et al., 2017b). On the other hand, when temperature treatments were applied at veraison, high temperatures induced a higher proportion of 3′4′5′-substituted anthocyanins (Tarara et al., 2008). In the current study, temperature treatments were applied at the same time (veraison) in all experiments; however, small differences in the timing of treatment applications may have occurred and resulted in the inconsistent results observed for the 3′4′5′-substituted proportion in the response to the treatments among experiments. It is noteworthy that, in Experiment 2, despite that the proportion of 3′4′5′-substituted anthocyanin is higher in the high temperature regimes at harvest (135 DAA), it was not at 113 DAA (**Figure S5B**). This indicates that the effects of the temperature regimes on the anthocyanin profile can change depending on the developmental stage of the grapes.

Previous studies suggested that a larger difference between day/night temperatures, due to lower night temperatures, favoured anthocyanin accumulation (Mori et al., 2005; Gaiotti et al., 2018). In our study, it has been observed that comparing the regimes with the same day but different night temperatures, the difference between day/night temperatures has no effect on either anthocyanin and flavonol accumulation or their profiles, except for flavonol levels that are higher in the 20/15 than in the 20/10 berries in Experiment 1. Remarkably, the difference between day/night temperatures affects anthocyanin levels, only when a day/night temperature difference of 25°C (30/5, in Experiment 3) is applied, compared with day/night temperature differences of 5°C (30/25) or 15°C (20/15). When the grapevines are subjected to regimes with the same night but different day temperatures, an effect on anthocyanin and flavonol accumulation is observed. The lack of effects of day/night temperature differences when the same day temperature is considered (except for 20/10 *vs.* 20/15 for flavonols in Experiment 1 and 30/5 *vs.* 30/15 and 30/25 for anthocyanins in Experiment 3), as well as the fact that within the regimes that have the same night temperature, anthocyanins, and flavonols change according to day temperatures, suggest that day temperature has a stronger effect than night temperature on anthocyanin and flavonol biosynthesis or/and degradation. A stronger effect of day than night temperature on anthocyanin accumulation was recently postulated by Gouot et al. (2018).

Previous studies have shown a circadian control on the expression of the flavonoid pathway genes and related TFs in Arabidopsis seeds (Harmer et al., 2000). In our study, out of 14 genes tested, six (*VviF3*′*5*′*Hf*, *VviFLS5*, *VviUFGT*, *VviAM3*, *VviMybA*, and *VviAOMT*) are affected by the sampling time (day *vs.* night) regardless of the temperature regimes and most of the genes that change in their expression between day/night have higher expression levels at night. Rienth et al. (2014b) showed that the expression pattern of the flavonoid pathway genes was distinct between day/night at veraison, but only a few showed consistent patterns at other developmental stages. Our study focuses on the expression analysis of two developmental stages (76 and 89 DAA), and clear trends are observed only for the six genes reported above; further studies that consider more developmental stages during fruit ripening are required to better understand a potential circadian regulation of the expression of flavonoid genes and its impact on flavonoid biosynthesis.

**Figure 3:**
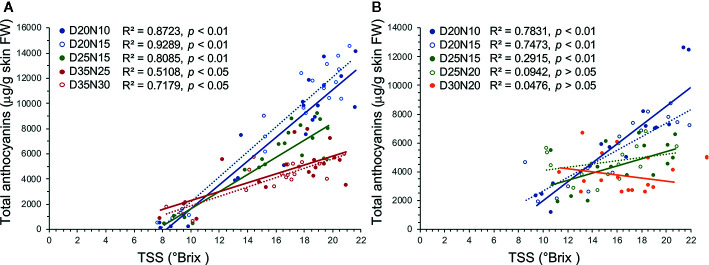
Linear regression analysis between sugar and anthocyanin accumulation during berry ripening in Merlot grapes exposed to different temperature regimes in Experiments 1 **(A)** and 2 **(B)**.

TSS and TA level are useful indicators of grape ripeness and quality. Overall, TSS levels at harvest were lower under high temperature regimes as in Kliewer (1977) and Mori et al. (2005), although the differences were not statistically significant in Experiment 1. Sugars regulate the anthocyanin accumulation in grape berry, and increased sugar level at veraison was reported to trigger the anthocyanin biosynthesis (Dai et al., 2014). Linear regression analysis reveals that there is a decoupling between sugar and anthocyanin levels under the high temperature regimes (**Figure 3**). There was a much more distinct decoupling of TSS and anthocyanin accumulation in regimes with the same night but different day temperatures, whereas this decoupling was absent or minor between regimes with the same day but different night temperatures (**Figure 3**, **Tables S7** and **S8**). Sadras and Moran (2012; 2013) reported that the decoupling of anthocyanin and sugar accumulation under elevated temperatures (1–3°C increase) during early stages of berry development was due to a delayed onset of anthocyanin accumulation during berry ripening. However, the decoupling of TSS and anthocyanin accumulation observed in the current study might be either the result of a lower rate of anthocyanin accumulation and/or a higher rate of anthocyanin degradation in the high temperature regimes (**Figure 3**). Arrizabalaga et al. (2018) suggested that the lower anthocyanin levels in grapes grown under high day/night temperature conditions (28/18°C compared with 24/14°C) were due to a relatively lower rate rather than a delayed rate of anthocyanin accumulation.

In this study, the TA decrease during berry ripening was stronger under high temperature regimes. The TA decrease during ripening is normally attributed to the degradation of malic acid which is increased by elevated temperatures (Ruffner et al., 1976; Bergqvist et al., 2001; Rienth et al., 2016). Sweetman et al. (2014) showed an increased sensitivity of malate metabolism to heating treatments during the day than at night. Consistently, in this study, regimes with the same high day temperature and different night temperatures resulted in similar TA levels (**Table 1**; **Figure S1**).

In conclusion, the application of the temperature regimes from veraison to harvest strongly affected anthocyanin and flavonol accumulation and shifted their profiles. Lower temperatures promoted anthocyanin and flavonol accumulation. Despite our initial hypothesis, no or limited effects of the difference between day/night temperatures were observed on anthocyanins and flavonols when regimes with the same day but different night temperatures were compared. On the contrary, when regimes with the same night but different day temperatures were compared, it was observed that a larger difference between day/night temperatures decreased the anthocyanin levels but increased the relative concentration of methoxylated and acylated anthocyanins as well as methoxylated and 3′4′5′-substituted flavonols. Consequently, we conclude that day temperature exerts stronger effects than night temperature on anthocyanin and flavonol accumulation in Merlot grapes. Despite the gene expression analysis was conducted only in one of the three experiments, this study reveals inconsistent responses to the temperature regimes among anthocyanin genes, which indicates that the major effect observed on anthocyanin levels and profiles might result from anthocyanin degradation at high temperatures and from a different rate of degradation among anthocyanin species. Conversely, the effect of the temperature regimes on flavonol levels was consistent with that observed on the levels of transcripts of key flavonol genes, thus suggesting a major regulatory mechanism at the transcriptional level. Gene expression was assessed only at two developmental stages that corresponded to the maximum rates of anthocyanin accumulation (**Figure 1**) and highest gene expression of anthocyanin related genes (Castellarin et al., 2007; Castellarin and Di Gaspero, 2007); however, the expression levels at other ripening stages might also have affected the anthocyanin levels and profiles at harvest. Molecules previously reported as final products of anthocyanin degradation were detected but they could not be quantified. For these reasons, further studies will be aimed on integrating a comprehensive characterization of gene expression profiles, enzyme activities, and the evolution of degradation products during grape ripening.

## Data Availability Statement

All data sets presented in this study are included in the article/**Supplementary Material**.

## Author Contributions

YY performed all the experiments and drafted the manuscript. CS collaborated with YY in performing the greenhouse and lab experiments. LF contributed to the data analysis and critically reviewed the manuscript. SC designed the study, analysed the data with YY and LF, and wrote the manuscript with YY. All authors contributed to the article and approved the submitted version.

## Funding

This research was supported by Natural Science and Engineering Research Council of Canada (NSERC RGPIN-2015-04760), the Canada Research Chair (950-230913) program, and China Scholarship Council (CSC).

## Conflict of Interest

The authors declare that the research was conducted in the absence of any commercial or financial relationships that could be construed as a potential conflict of interest.

## Supplementary Material

Figure S1 Temperature effects on total soluble solids (TSS, **A** and **B**) and titratable acidity (TA, **C** and **D**). Values reported are the mean ± standard error (SE, n = 4). Different letters indicate significantly different means according to an LSD test (*p* ≤ 0.05). Legend in **(A)** indicates the temperature regimes in **(A, C)**; legend in **(B)** indicates the temperature regimes in **(B, D)**. DAA refers to days after anthesis.

Figure S2 Temperature effects on anthocyanin **(A, B)** and flavonol **(C, D)** concentration (µg/g berry FW) in Merlot grapes in Experiments 1 **(A, C)** and 2 **(B, D)**. Values reported are the mean ± standard error (SE, n = 4). Different letters indicate significant different means according to an LSD test (*p* ≤ 0.05). Legend in **(A)** indicates the temperature regimes in (A, C); legend in **(B)** indicates the temperature regimes in B and D. DAA refers to days after anthesis.

Figure S3 Temperature effects on anthocyanin **(A, B)** and flavonol **(C, D)** content (µg/berry) in Merlot grapes in Experiments 1 **(A, C)** and 2 **(B, D)**. Values reported are the mean ± standard error (SE, n = 4). Different letters indicate significant different means according to an LSD test (*p* ≤ 0.05). Legend in **(A)** indicates the temperature regimes in **(A, C)**; legend in **(B)** indicates the temperature regimes in **(B, D)**. DAA refers to days after anthesis.

Figure S4 Temperature effects on the evolution of the relative concentration of acylated anthocyanins during berry ripening in Experiments 1 **(A, C, E)** and 2 **(B, D, F)**. Values represent the mean ± standard error (SE, n = 4). Different letters indicate significantly different means according to an LSD test (*p* ≤ 0.05). Legend in **(A)** indicates the temperature regimes in **(A, C, E)**; legend in **(B)** indicates the temperature regimes in **(B, D, F)**. DAA refers to days after anthesis.

Figure S5 Temperature effects on the evolution of the relative concentration of 3′4′5′-substituted **(A, B)** and methoxylated **(C, D)** anthocyanins during berry ripening in Experiments 1 **(A, C)** and 2 **(B, D)**. Values represent the mean ± standard error (SE, n = 4). Different letters represent significantly different means according to an LSD test (*p* ≤ 0.05). Legend in **(A)** indicates the temperature regimes in **(A, C)** legend in **(B)** indicates the temperature regimes in **(B, D)**. DAA refers to days after anthesis.

Figure S6 Temperature effects on the evolution of the relative concentration of differentially substituted flavanols during berry ripening in Experiment 1 **(A, C, E)** and 2 **(B, D, F)**. Values represent the mean ± standard error (SE, n = 4). Different letters represent significantly different means according to an LSD test (*p* ≤ 0.05). Legend in **(A)** indicates the temperature regimes in **(A, C, E)**; legend in **(B)** indicates the temperature regimes in **(B, D, F)**. DAA refers to days after anthesis.

Figure S7 Temperature effects on the evolution of the relative concentration of methoxylated flavonols during berry ripening in Experiment 1 **(A)** and 2 **(B)**. Values represent the mean ± standard error (SE, n = 4). Different letters represent significantly different means according to an LSD test (*p* ≤ 0.05). DAA refers to days after anthesis.

Figure S8 Mass spectra of authentic standards of anthocyanin degradation products (upper spectrum of each panel) and anthocyanin degradation products detected in grape samples (lower spectrum of each panel) by LC-MS/MS analysis (negative ESI) using an LC-QTOF. **(A)** gallic acid; **(B)** protocatechuic acid; **(C)** 4-hydroxybenzoic acid; **(D)** syringic acid.

## Abbreviations

PAL, phenylpropanoid ammonium lyase; CHS, chalcone synthase; CHI, chalcone isomerase; UFGT, UDP-glucose: flavonoid 3-O-glucosyltransferase; TF, transcription factor; RH, relative humidity; PAR, photosynthetically active radiation; DAA, days after anthesis; TSS, total soluble solids; TA, titratable acidity; HPLC, high-performance liquid chromatography; MS, mass spectrometry; F3′5′H, flavonoid 3′5′-hydroxylase; FLS, flavonol synthase; AM3, anthoMATE3; GST, glutathione S-transferase; F3′H, flavonoid 3′-hydroxylase; LDOX, leucoanthocyanidin dioxygenase; AOMT, anthocyanin O-methyl transferase; 3AT, anthocyanin 3-O-glucoside 6′′-O-acyltransferase
