# Supplementary material for: Day Temperature Has a Stronger Effect Than Night Temperature on Anthocyanin and Flavonol Accumulation in ‘Merlot’ (Vitis vinifera L.) Grapes During Ripening
Source: Front Plant Sci. 2020 Jul 24;11:1095. doi: 10.3389/fpls.2020.01095 (PMC7396706; doi:10.3389/fpls.2020.01095)
Supplement: Supplementary file 1 [file Table_1.pdf]

Table S1 Description of the temperature regimes in Experiments 1, 2, and 3. DAA refers to days after anthesis.

Experiment	Temperature regimes (day/night temperature, °C)	Arbitrary name (based on day temperature)	Duration of temperature regimes applied (start-end DAA, days of application)
Experiment 1	20/10	Low temperature regime	60-118, 58
	20/15	Low temperature regime	60-118, 58
	25/15	Intermediate temperature regime	60-118, 58
	35/25	High temperature regime	60-118, 58
	35/30	High temperature regime	60-118, 58
Experiment 2	20/10	Low temperature regime	69-135, 66
	20/15	Low temperature regime	69-135, 66
	25/15	Intermediate temperature regime	69-135, 66
	25/20	Intermediate temperature regime	69-135, 66
	30/20	High temperature regime	69-135, 66
Experiment 3	20/5	Low temperature regime	67-113, 46
	20/15	Low temperature regime	67-113, 46
	30/5	High temperature regime	67-113, 46
	30/15	High temperature regime	67-113, 46
	30/25	High temperature regime	67-113,46

Table S2 Forward (for) and reverse (rev) primers of genes selected for quantitative real time PCR (qRT-PCR)

Gene name	Primer sequence_for	Primer sequence_ rev	Reference
VviUbiquitin1	GTGGTATTATTGAGCCATCCTT	AACCTCCAATCCAGTCATCTAC	Bogs et al. (2006)
VviCHI1	CAGGCAACTCCATTCTTTTC	TTCTCTATCACTGCATTCCC	Azuma et al. (2012)
VviF3'Ha	GGCGGAAGGTTTCCTTGAT	GCACGTTGATCTCGGTGAG	Falginella et al. (2010)
VviF3'5'Hf	TGTACCAACGACCCCAAAAT	GAACCTTCCTCGTGTCTCAG	Falginella et al. (2010)
VviF3'5'Hi	GCCAGAGACCACTCGATTAC	ACCCAGATTTTCTGGACGTG	Falginella et al. (2010)
VviFLS4	AAACCACCTACTTACAGAGC	ACCTAACCCCAGTGACAGAC	Fujita et al. (2006)
VviFLS5	AACCAAGATGACTAAGAACC	CTTCTGTGACTTCCCTGTAG	Fujita et al. (2006)
VviLDOX	AGGGAAGGGAAAACAAGTAG	ACTCTTTGGGGATTGACTGG	Azuma et al. (2012)
VviUFGT	AATCTGAGAGCCCTAAGAGA	GGTGGTACAAGCAACAGTTC	Movahed et al. (2016)
VviAOMT	CTCTGCAGGCGCCTCTATTA	CCCAAAACAGAGTCTGGACA	Hugueney et al. (2009)
Vvi3AT	AGTGAGTCGCGAGGATGTGTTGT	TCCAAGCAGGATTTCCCCAACCA	Rinaldo et al. (2015)
VviGST4	ACTTGGTGAAGGAAGCTGGA	TTGGAAAGGTGCATACATGG	Terrier et al. (2005)
VviAM3	GCAAACAACAGAGAGGATGC	AGACCTCGACAATGATCTTAC	Gomez et al. (2009)
VviMybA	GAGGGTGATTTTCCATTTGAT	CAAGAACAACTTTTGAACTTAAACAT	Bogs et al. (2006)
VviMybF1	GGAGGTTGAGGGGTTGTG	AAGTTGGGGAAGAGCAGGAG	Czemmel et al. (2009)

Table S3 Temperature effects on berry development at harvest in Experiments 1, 2, and 3. Values reported are the mean ± standard error (SE, n = 4). Different letters indicate significantly different means within each column in each experiment according to an LSD test ($p \leq 0.05$); DAA refers to days after anthesis.

Experiment	Temperature regimes (day/night temperature, °C)	Average berry weight (g)	Average skin weight (mg)	Average seed weight (mg)	Skin to berry weight ratio
Experiment 1 (118 DAA)	20/10	1.09 ± 0.07 ab	98.84 ± 7.17 a	38.74 ± 2.97	0.091 ± 0.003
	20/15	1.19 ± 0.07 ab	97.31 ± 8.72 a	38.33 ± 2.22	0.082 ± 0.006
	25/15	1.29 ± 0.09 a	85.06 ± 7.64 b	41.09 ± 1.22	0.066 ± 0.005
	35/25	0.96 ± 0.06 b	85.09 ± 10.79 b	36.97 ± 2.32	0.086 ± 0.011
	35/30	0.93 ± 0.13 b	64.88 ± 2.94 c	39.82 ± 2.88	0.069 ± 0.012
Experiment 2 (135 DAA)	20/10	1.15 ± 0.13	110.40 ± 4.68 a	39.37 ± 1.67	0.097 ± 0.008 a
	20/15	0.98 ± 0.05	97.33 ± 4.58 ab	37.74 ± 2.09	0.099 ± 0.006 a
	25/15	1.28 ± 0.013	97.61 ± 2.16 ab	37.91 ± 2.01	0.076 ± 0.002 b
	25/20	1.28 ± 0.19	100.94 ± 4.12 ab	37.52 ± 2.54	0.079 ± 0.001 b
	30/20	1.12 ± 0.12	77.87 ± 3.46 b	37.20 ± 1.31	0.070 ± 0.003 b
Experiment 3 (113 DAA)	20/5	1.27 ± 0.07	113.91 ± 9.37 ab	41.2 ± 2.59	0.091 ± 0.01 a
	20/15	1.28 ± 0.05	118.72 ± 8.72 a	36.31 ± 1.16	0.093 ± 0.004 a
	30/5	1.29 ± 0.1	92.66 ± 6.75 b	39.78 ± 1.14	0.072 ± 0.002 ab
	30/15	1.43 ± 0.06	95.83 ± 9.6 ab	38.74 ± 0.94	0.067 ± 0.006 b
	30/25	1.28 ± 0.06	101.4 ± 5.72 ab	37.83 ± 0.95	0.079 ± 0.003 ab

Table S4 The identification of anthocyanin and flavonol compounds in Experiments 1, 2, and 3.

No.	Compound	Retention time (min)	*m/z* ($M+H^+$)	Mass loss [($M+H^+$)-MS^2]	MS^2
1	Delphinidin 3-O-glucoside	2.2	465	162	303
2	Cyanidin 3-O-glucoside	2.9	449	162	287
3	Petunidin 3-O-glucoside	3.4	479	162	317
4	Peonidin 3-O-glucoside	3.9	463	162	301
5	Malvidin 3-O-glucoside	4.4	493	162	331
6	Delphinidin 3-O-(6"-acetyl)glucoside	4.6	507	204	303
7	Cyanidin 3-O-(6"-acetyl)glucoside	5.2	491	204	287
8	Petunidin 3-O-(6"-acetyl)glucoside	5.6	521	204	317
9	Peonidin 3-O-(6"-acetyl)glucoside	6.3	505	204	301
10	Malvidin 3-O-(6"-acetyl)glucoside	6.5	535	204	331
11	Dephinidin 3-O-(6"-*p*-coumaroyl)glucoside	6.2	611	308	303
12	Cyanidin 3-O-(6"-*p*-coumaroyl)glucoside	6.8	595	308	287
13	Petunidin 3-O-(6"-*p*-coumaroyl)glucoside	7.0	625	308	317
14	Peonidin 3-O-(6"-*p*-coumaroyl)glucoside	7.4	609	308	301
15	Malvidin 3-O-(6"-*p*-coumaroyl)glucoside	7.5	639	308	331
1	Myricetin 3-O-galactoside	3.8	479	162	317
2	Myricetin 3-O-glucoside	3.9	479	162	317
3	Myricetin 3-O-glucuronide	4.1	493	176	317
4	Quercetin 3-O-galactoside	4.7	463	162	301
5	Quercetin 3-O-glucuronide	4.8	477	176	301
6	Quercetin 3-O-glucoside	4.9	463	162	301
7	Kaempferol (acetyl)glucoside	5.1	489	204	285
8	Kaempferol 3-O-glucoside	5.8	447	162	285
9	Quercetin (ramnosyl)glucoside	6.0	609	308	301
10	Isorhamnetin 3-O-glucoside	6.3	477	162	315
11	Syringetin 3-O-glucoside	6.5	507	162	345

Table S5 The identification of anthocyanin degradation products

No.	Compound *	Retention time (min)	*m/z* (M-H^+)	Qualifying ions
1	Gallic acid	0.8	169	108, 95
2	Protocatechuic acid	1.2	153	108, 65
3	4-Hydroxybenzoic acid	1.8	137	118, 99
4	Syringic acid	3.7	197	162

* Anthocyanin degradation products were detected by LC-MS/MS (negative ESI) using an LC-QTOF and identified in the samples tested (20/10, 20/15, 35/25, 35/30 samples in Experiment 1) by matching the target ion and retention time with authentic standards (gallic acid, protocatechuic acid, 4-hydroxybenzoic acid, and syringic acid). The concentrations were lower than limit of quantification (LOQ).

Table S6 Light intensity at the cluster level in Experiment 1, 2, and 3, and berry surface temperature in Experiment 1 and 3. Values reported are the mean ± standard error (SE, n = 6 for light intensity and n = 4 for berry surface temperature).

	Temperature regimes (day/night temperature, °C)				
	20/10	20/15	25/15	35/25	35/30
Light intensity at the cluster (μmol m^{-2} s^{-1} of PAR)	52.5 ± 9.4	46.7 ± 12.9	44.2 ± 3.3	53.3 ± 6.5	51.8 ± 5.3
Berry surface temperature day (°C)	20.5 ± 0 d	20.2 ± 0.1 d	25.1 ± 0.1 c	35.1 ± 0.1 a	34.4 ± 0.1 b
Berry surface temperature night (°C)	9.7 ± 0.1 e	14.6 ± 0 d	15.5 ± 0 c	24.4 ± 0.2 b	30.0 ± 0.1 a
	20/10	20/15	25/15	25/20	30/20
Light intensity at the cluster (μmol m^{-2} s^{-1} of PAR)	43.3 ± 15.8	29.4 ± 5.9	26.9 ± 11.4	22.8 ± 8.4	28.7 ± 6.6
	20/5	20/15	30/5	30/15	30/25
Light intensity at the cluster (μmol m^{-2} s^{-1} of PAR)	25.5 ± 2.5	27.0 ± 2.4	28.4 ± 3.2	28.6 ± 2.8	27.2 ± 3.0
Berry surface temperature day (°C)	20.1 ± 0.1 c	19.6 ± 0.2 d	29.5 ±0.1 ab	29.3 ± 0.1 b	29.7 ± 0.1 a
Berry surface temperature night (°C)	4.8 ± 0.1 c	15.0 ± 0.1 b	4.8 ± 0.1 c	15.2 ± 0.1 b	24.7 ± 0.1 a

Table S7 *p* values of the ANCOVA analysis reported in **Figure 3A**. The equation of the regression lines are as listed: i) 20/10, y = 944.32x – 7798.6; ii) 20/15, y = 991.48x – 7728.7; iii) 25/15, y = 681.07x – 5141.7; iv) 35/25, y = 351.49x – 1226.6; v) 35/30, y = 372.17x – 1853.6.

p values of coefficients between temperature regimes	20/10	20/15	25/15	35/25	35/30
20/10	NA	0.432	0.005 **	< 0.001 **	< 0.001 **
20/15	0.432	NA	0.006 **	< 0.001 **	< 0.001 **
25/15	0.005 **	0.006 **	NA	< 0.001 **	< 0.001 **
35/25	< 0.001 **	< 0.001 **	< 0.001 **	NA	0.156
35/30	< 0.001 **	< 0.001 **	< 0.001 **	0.156	NA

Table S8 *p* values of the ANCOVA analysis reported in **Figure 3B**. The equation of the regression lines are as listed: i) 20/10, y = 653.22x – 4533.6; ii) 20/15, y = 470.89x – 2024.7; iii) 25/15, y = 248.48x – 439.39; iv) 35/25, y = 116.03x – 2903.6; v) 35/30, y = -111.53x + 5563.5.

p values of coefficients between temperature regimes	20/10	20/15	25/15	25/20	30/20
20/10	NA	< 0.001 **	< 0.001 **	< 0.001 **	< 0.001 **
20/15	< 0.001 **	NA	< 0.001 **	< 0.001 **	< 0.001 **
25/15	< 0.001 **	< 0.001 **	NA	< 0.001 **	< 0.001 **
25/20	< 0.001 **	< 0.001 **	< 0.001 **	NA	< 0.001 **
30/20	< 0.001 **	< 0.001 **	< 0.001 **	< 0.001 **	NA

References

Azuma, A., Yakushiji, H., Koshita, Y., & Kobayashi, S. (2012). Flavonoid biosynthesis-related genes in grape skin are differentially regulated by temperature and light conditions. *Planta*, *236*(4), 1067–1080. DOI: 10.1007/s00425-012-1650-x

Bogs, J., Ebadi, A., McDavid, D., & Robinson, S. P. (2006). Identification of the flavonoid hydroxylases from grapevine and their regulation during fruit development. *Plant Physiol.*, *140*(1), 279–291. DOI: 10.1104/pp.105.073262

Czemmel, S., Stracke, R., Weisshaar, B., Cordon, N., Harris, N. N., ..., & Bogs, J. (2009). The grapevine R2R3-MYB transcription factor *VvMYBF1* regulates flavonol synthesis in developing grape berries. *Plant Physiol.*, *151*(3), 1513–1530. DOI: 10.1104/pp.109.142059

Falginella, L., Castellarin, S. D., Testolin, R., Gambetta, G. A., Morgante, M., & Di Gaspero, G. (2010). Expansion and subfunctionalisation of flavonoid 3', 5'-hydroxylases in the grapevine lineage. *BMC Genomics*, *11*(1), 562. DOI: 10.1186/1471-2164-11-562

Fujita, A., Goto-Yamamoto, N., Aramaki, I., & Hashizume, K. (2006). Organ-specific transcription of putative flavonol synthase genes of grapevine and effects of plant hormones and shading on flavonol biosynthesis in grape berry skins. *Biosci. Biotech. Biochem.*, *70*(3), 632–638. DOI: 10.1271/bbb.70.632

Gomez, C., Terrier, N., Torregrosa, L., Vialet, S., Fournier-Level, A., …, & Ageorges, A. (2009). Grapevine MATE-type proteins act as vacuolar H^+-dependent acylated anthocyanin transporters. *Plant Physiol.*, *150*(1), 402–415. DOI: 10.1104/pp.109.135624

Hugueney, P., Provenzano, S., Verriès, C., Ferrandino, A., Meudec, E., Batelli, G., Merdinoglu, D., Cheynier, V., Schubert, A., & Ageorges, A. (2009). A novel cation-dependent O-methyltransferase involved in anthocyanin methylation in grapevine. *Plant Physiol.*, *150*(4), 2057–2070. DOI: 10.1104/pp.109.140376

Movahed, N., Pastore, C., Cellini, A., Allegro, G., Valentini, G., Zenoni, S., Cavallini, E., D'Incà, E., Tornielli, G. B., & Filippetti, I. (2016). The grapevine VviPrx31 peroxidase as a candidate gene involved in anthocyanin degradation in ripening berries under high temperature. *J. Plant Res.*, *129*(3), 513–526. DOI: 10.1007/s10265-016-0786-3

Rinaldo, A., Cavallini, E., Jia, Y., Moss, S. M. A., McDavid, D. A. J., …, & Walker, A. R. (2015). A grapevine anthocyanin acyltransferase, transcriptionally regulated by *VvMYBA*, can produce most acylated anthocyanins present in grape skins. *Plant Physiol.*, *169*(3), 1897–1916. DOI: 10.1104/pp.15.01255

Terrier, N., Glissant, D., Grimplet, J., Barrieu, F., Abbal, P., ..., & Dédaldéchamp, F. (2005). Isogene specific oligo arrays reveal multifaceted changes in gene expression during grape berry (*Vitis vinifera* L.) development. *Planta*, *222*(5), 832–847. DOI: 10.1007/s00425-005-0017-y
