# Supplementary figures and images for: Day Temperature Has a Stronger Effect Than Night Temperature on Anthocyanin and Flavonol Accumulation in ‘Merlot’ (Vitis vinifera L.) Grapes During Ripening
Source: Front Plant Sci. 2020 Jul 24;11:1095. doi: 10.3389/fpls.2020.01095 (PMC7396706; doi:10.3389/fpls.2020.01095)

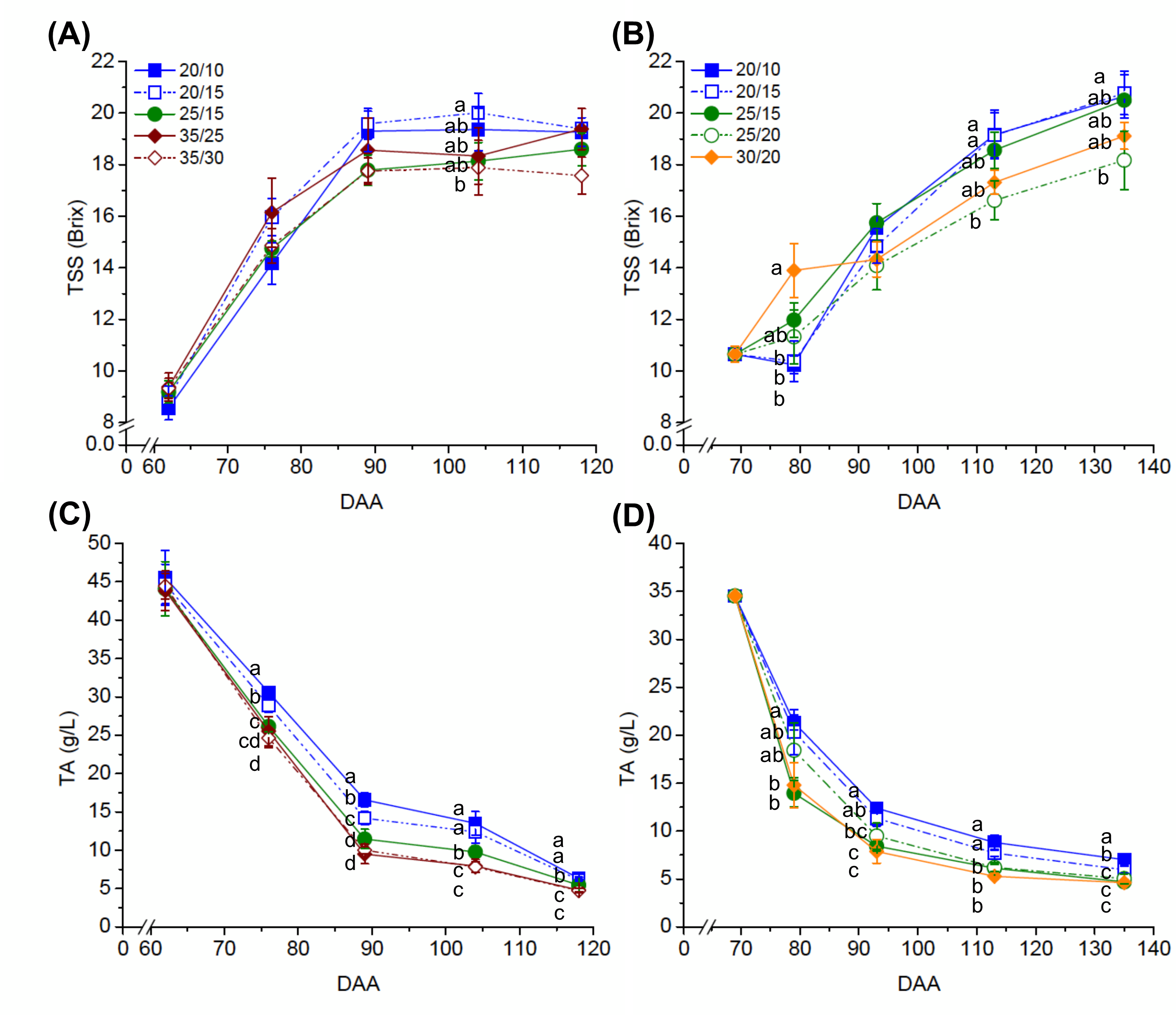

Supplement: Figure S1 — Temperature effects on total soluble solids (TSS, A and B) and titratable acidity (TA, C and D). Values reported are the mean ± standard error (SE, n = 4). Different letters indicate significantly different means according to an LSD test (p ≤ 0.05). Legend in (A) indicates the temperature regimes in (A, C); legend in (B) indicates the temperature regimes in (B, D). DAA refers to days after anthesis. [file Image_1.tiff]

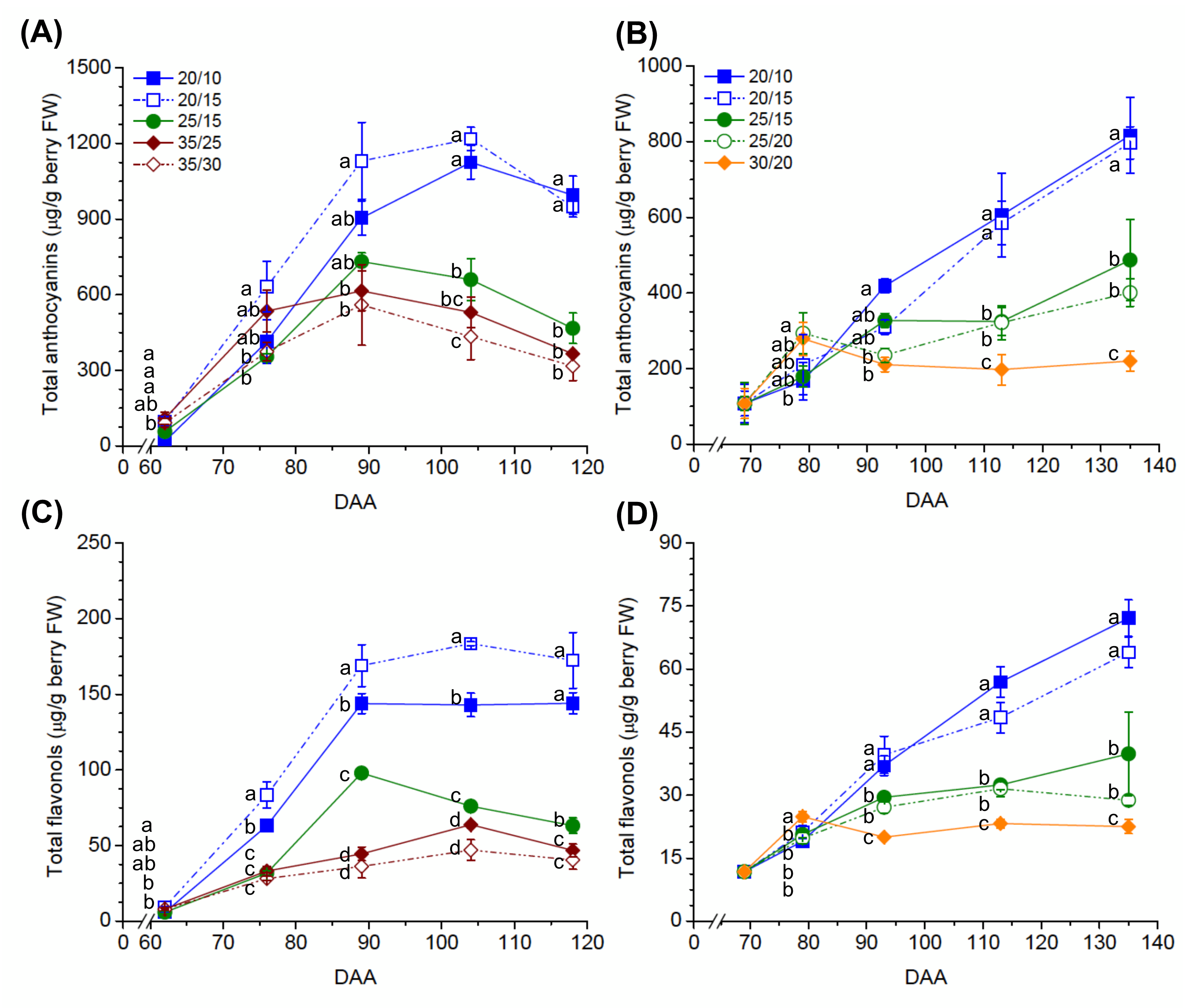

Supplement: Figure S2 — Temperature effects on anthocyanin (A, B) and flavonol (C, D) concentration (µg/g berry FW) in Merlot grapes in Experiments 1 (A, C) and 2 (B, D). Values reported are the mean ± standard error (SE, n = 4). Different letters indicate significant different means according to an LSD test (p ≤ 0.05). Legend in (A) indicates the temperature regimes in (A, C); legend in (B) indicates the temperature regimes in B and D. DAA refers to days after anthesis. [file Image_2.tiff]

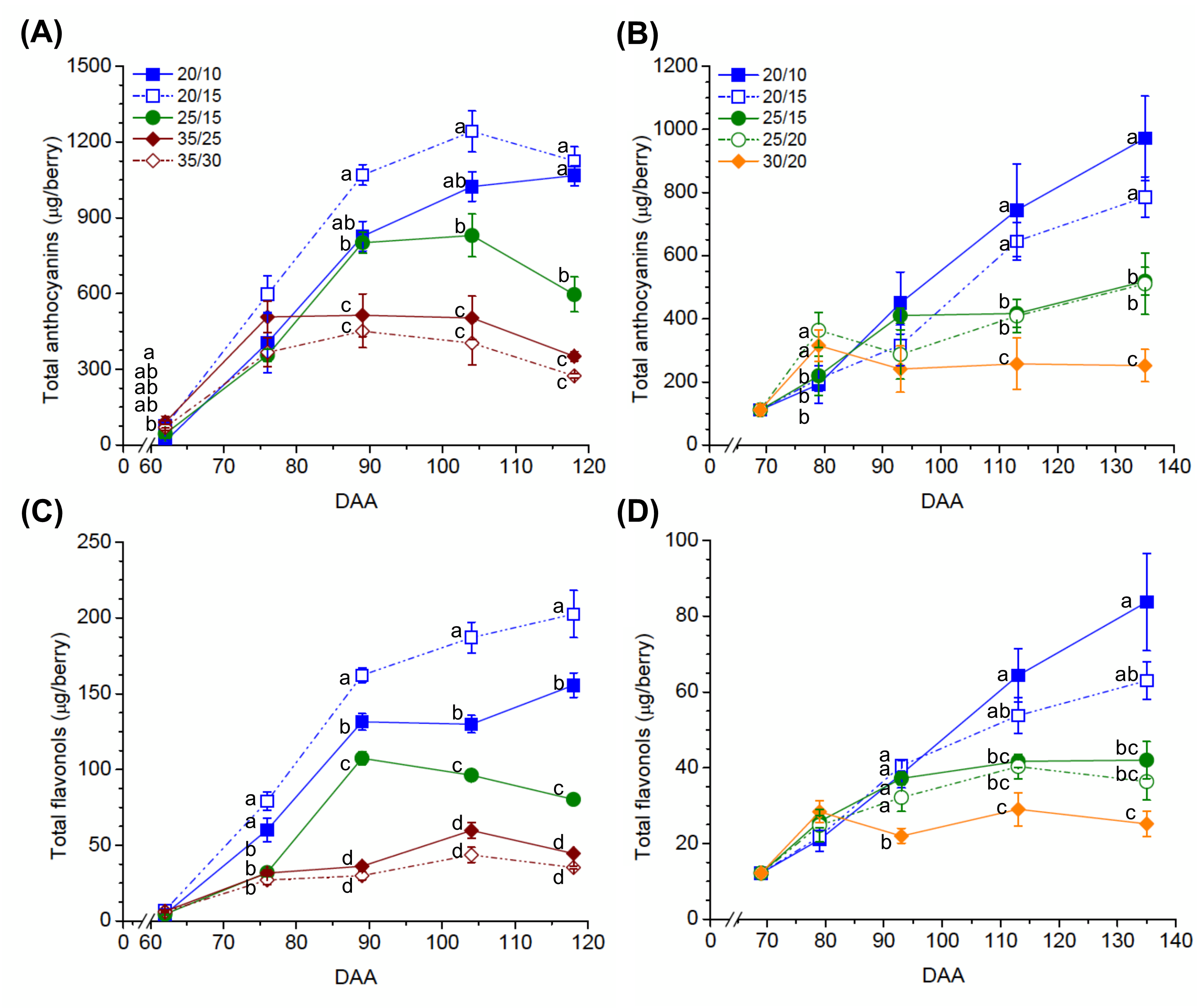

Supplement: Figure S3 — Temperature effects on anthocyanin (A, B) and flavonol (C, D) content (µg/berry) in Merlot grapes in Experiments 1 (A, C) and 2 (B, D). Values reported are the mean ± standard error (SE, n = 4). Different letters indicate significant different means according to an LSD test (p ≤ 0.05). Legend in (A) indicates the temperature regimes in (A, C); legend in (B) indicates the temperature regimes in (B, D). DAA refers to days after anthesis. [file Image_3.tiff]

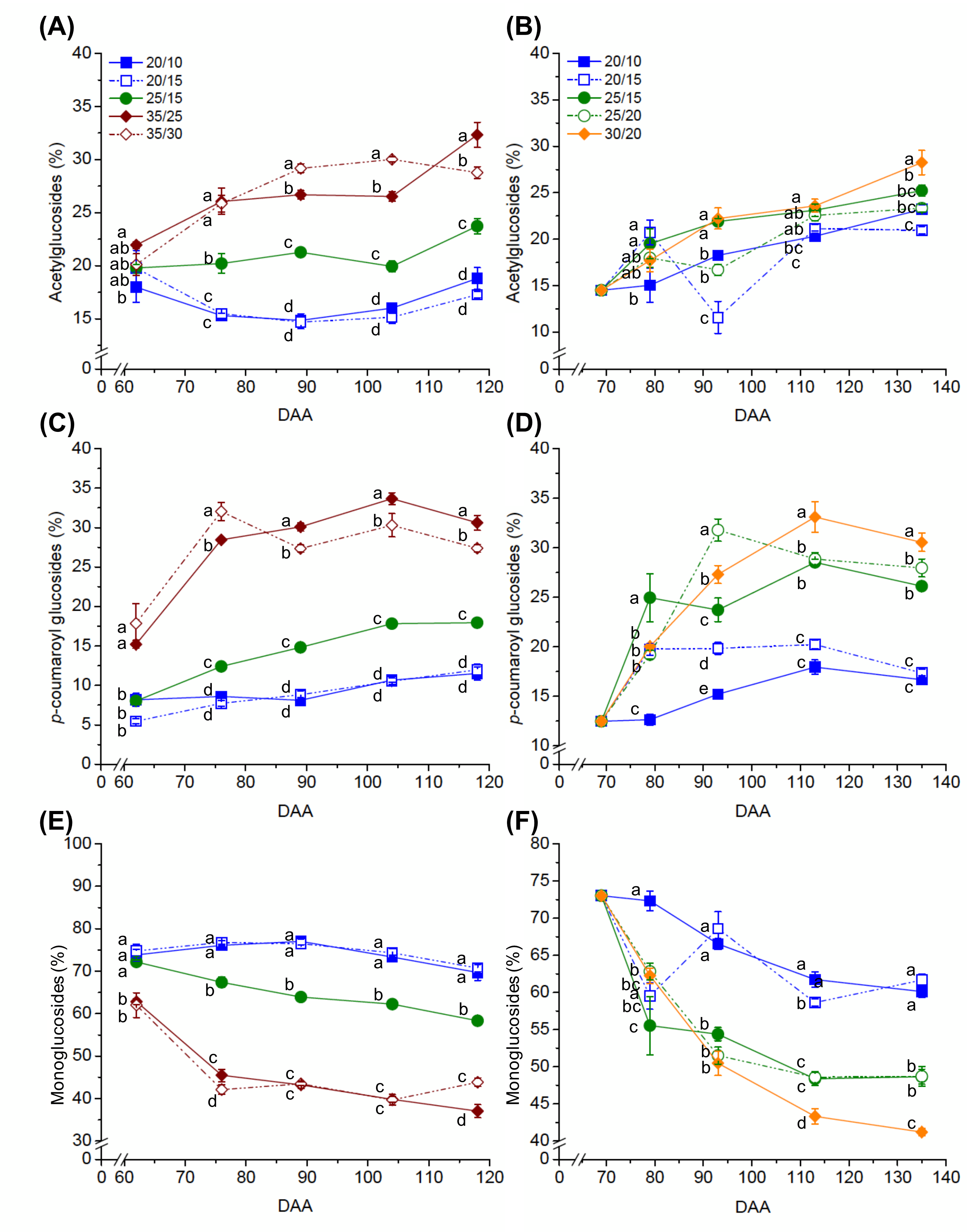

Supplement: Figure S4 — Temperature effects on the evolution of the relative concentration of acylated anthocyanins during berry ripening in Experiments 1 (A, C, E) and 2 (B, D, F). Values represent the mean ± standard error (SE, n = 4). Different letters indicate significantly different means according to an LSD test (p ≤ 0.05). Legend in (A) indicates the temperature regimes in (A, C, E); legend in (B) indicates the temperature regimes in (B, D, F). DAA refers to days after anthesis. [file Image_4.tiff]

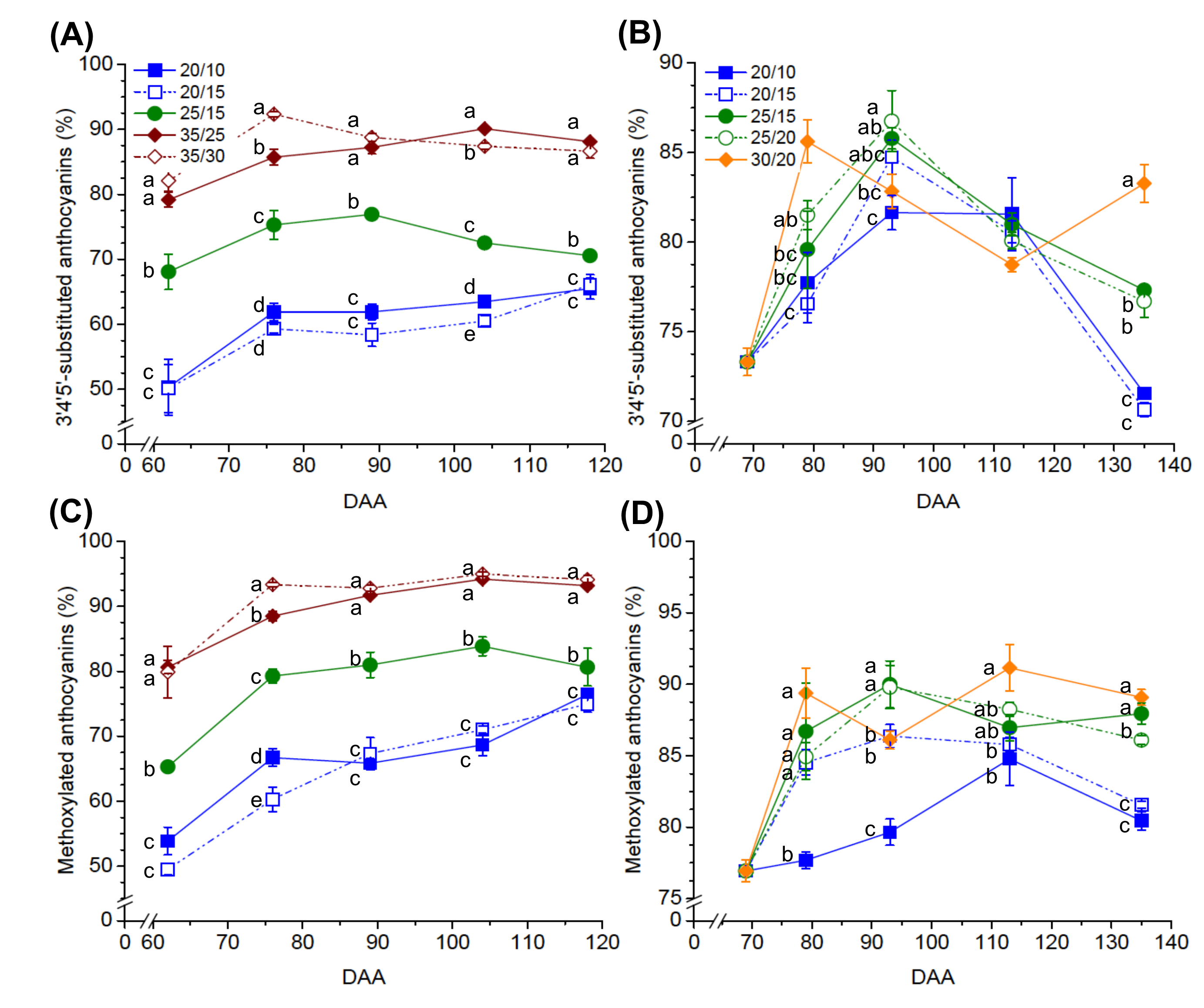

Supplement: Figure S5 — Temperature effects on the evolution of the relative concentration of 3′4′5′-substituted (A, B) and methoxylated (C, D) anthocyanins during berry ripening in Experiments 1 (A, C) and 2 (B, D). Values represent the mean ± standard error (SE, n = 4). Different letters represent significantly different means according to an LSD test (p ≤ 0.05). Legend in (A) indicates the temperature regimes in (A, C) legend in (B) indicates the temperature regimes in (B, D). DAA refers to days after anthesis. [file Image_5.tiff]

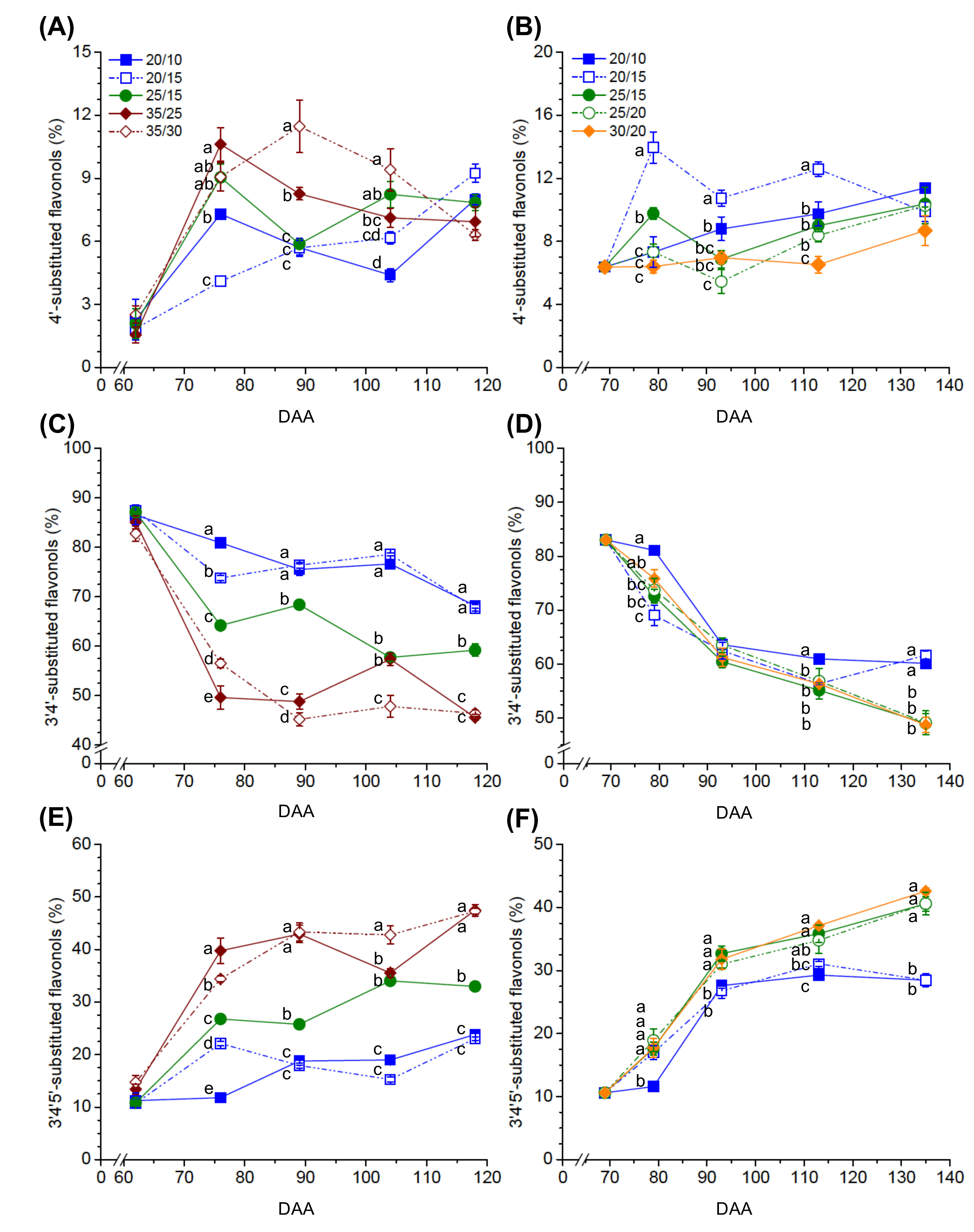

Supplement: Figure S6 — Temperature effects on the evolution of the relative concentration of differentially substituted flavanols during berry ripening in Experiment 1 (A, C, E) and 2 (B, D, F). Values represent the mean ± standard error (SE, n = 4). Different letters represent significantly different means according to an LSD test (p ≤ 0.05). Legend in (A) indicates the temperature regimes in (A, C, E); legend in (B) indicates the temperature regimes in (B, D, F). DAA refers to days after anthesis. [file Image_6.tiff]

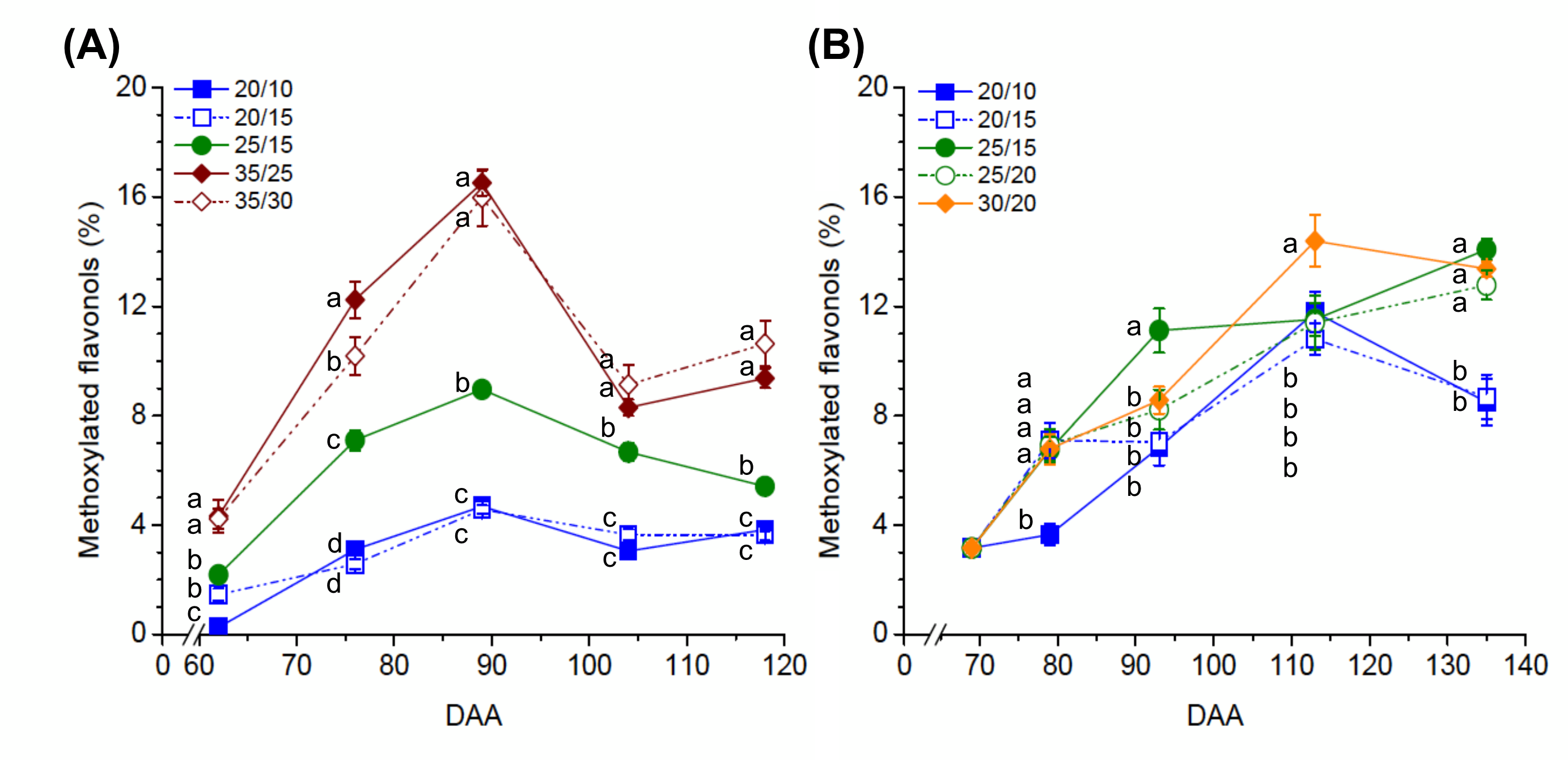

Supplement: Figure S7 — Temperature effects on the evolution of the relative concentration of methoxylated flavonols during berry ripening in Experiment 1 (A) and 2 (B). Values represent the mean ± standard error (SE, n = 4). Different letters represent significantly different means according to an LSD test (p ≤ 0.05). DAA refers to days after anthesis. [file Image_7.tiff]

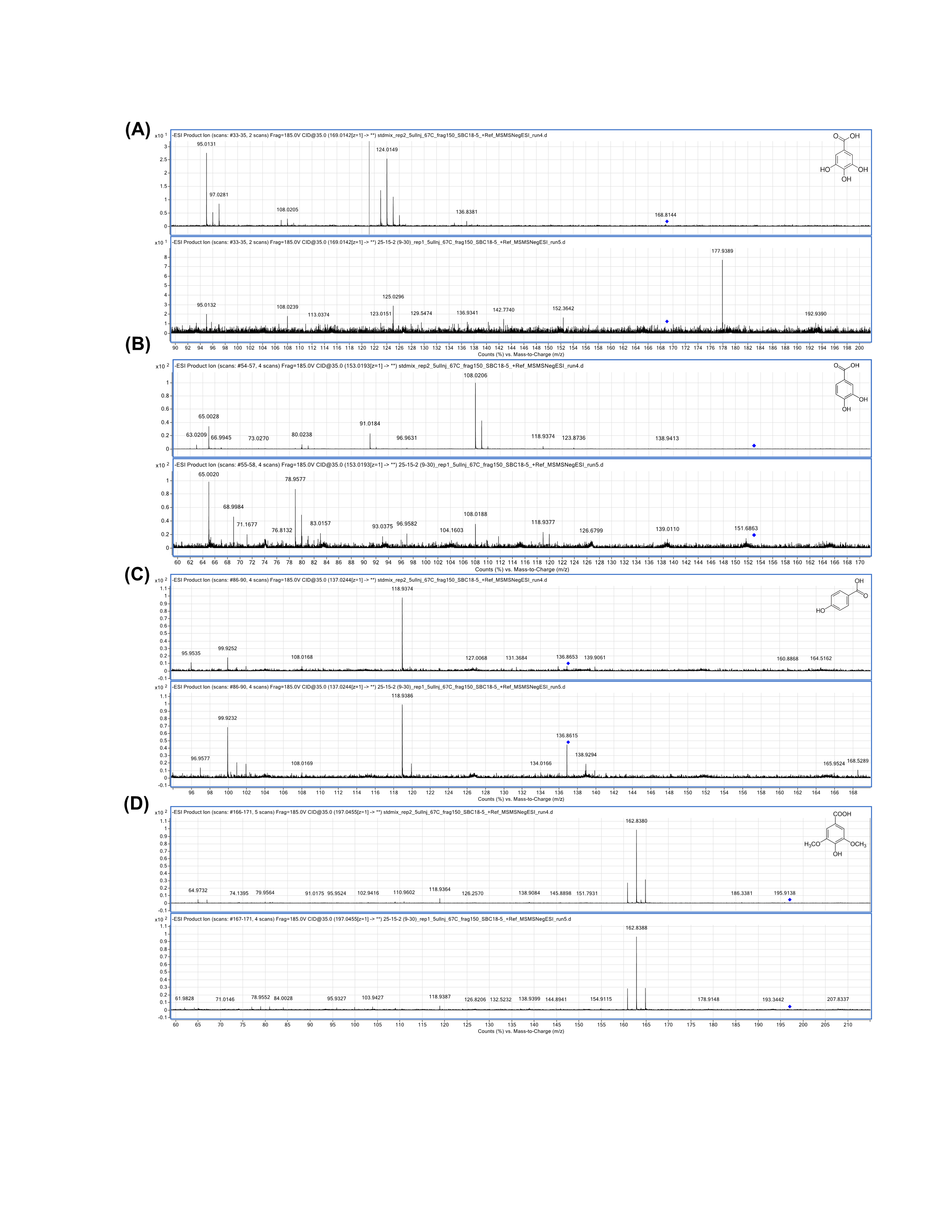

Supplement: Figure S8 — Mass spectra of authentic standards of anthocyanin degradation products (upper spectrum of each panel) and anthocyanin degradation products detected in grape samples (lower spectrum of each panel) by LC-MS/MS analysis (negative ESI) using an LC-QTOF. (A) gallic acid; (B) protocatechuic acid; (C) 4-hydroxybenzoic acid; (D) syringic acid. [file Image_8.tiff]
